# Assessing the nature of the charge-transfer electronic states in organic solar cells

**DOI:** 10.1038/s41467-018-07707-8

**Published:** 2018-12-13

**Authors:** Xian-Kai Chen, Veaceslav Coropceanu, Jean-Luc Brédas

**Affiliations:** School of Chemistry and Biochemistry and Center for Organic Photonics and Electronics, Georgia Institute of Technology, Atlanta, Georgia 30332-0400 USA

## Abstract

The charge-transfer electronic states appearing at the donor-acceptor interfaces in organic solar cells mediate exciton dissociation, charge generation, and charge recombination. To date, the characterization of their nature has been carried out on the basis of models that only involve the charge-transfer state and the ground state. Here, we demonstrate that it is essential to go beyond such a two-state model and to consider explicitly as well the electronic and vibrational couplings with the local absorbing state on the donor and/or acceptor. We have thus developed a three-state vibronic model that allows us: to provide a reliable description of the optical absorption features related to the charge-transfer states; to underline the erroneous interpretations stemming from the application of the semi-classical two-state model; and to rationalize how the hybridization between the local-excitation state and charge-transfer state can lead to lower non-radiative voltage losses and higher power conversion efficiencies.

## Abstract (web summary)

Previous descriptions of the charge-transfer absorptions in organic solar cells only involve the charge transfer state and the ground state. Here Chen et al. underline that a third state, *i*.*e*., the local absorbing state on the donor and/or acceptor, needs to be considered.

## Introduction

The most efficient organic solar cells (OSC) based on blends of conjugated polymers or oligomers (electron donors) and (non-)fullerene derivatives (electron acceptors) currently reach power conversion efficiencies (PCE) up to 14–15% in single-junction devices^1,2^ and 17% in tandem cells^3^, values that, however, remain significantly lower than in crystalline silicon^4^ or perovskite solar cells^5^. In OSCs, the excitons photogenerated in the donor (D) and/or acceptor (A) components dissociate at the D/A interfaces. This process results in either immediate long-range charge separation or formation of interfacial charge-transfer (CT) states; the nongeminate (bimolecular) recombination of initially separated charges also results in the formation of CT states. These interfacial CT states can either separate into free charges or recombine to the electronic ground state via radiative or non-radiative pathways. Thus, with respect to the energy of the local excitonic states on the absorbing species, there exist two significant sources of *V*_OC_ (energy) losses, related to exciton dissociation and charge recombination that both are mediated by the CT states^6–14^. Thus, given the critical role played by these CT states, it is crucial to develop robust methodologies that provide reliable information on their electronic and optical characteristics.

In most OSC materials, the CT absorptions are very weak, which means that highly sensitive photo-thermal deflection spectroscopy (PDS) or Fourier-transform photocurrent spectroscopy (FTPS) techniques are required to detect the CT states^7,15^. Then, extracting the electronic-structure information from the CT absorption bands has been commonly performed by analyzing the low-energy tail of the measured PDS or FTPS spectra in the framework of the semi-classical two-state Mulliken–Hush model. Importantly, this model exclusively considers the ground (G) electronic state and the full electron-transfer (Coulomb-bound electron–hole pair) state, which we refer to here as the D^+^A^−^ state^16,17^; it completely neglects any potential role played by the strongly absorbing local-excitation (LE) state on the donor and/or acceptor, even though the LE and D^+^A^−^ states have to be electronically coupled in order for exciton dissociation to take place.

Recently, in order to reduce *V*_OC_ losses, a large number of experimental efforts have been devoted to develop D/A active layers in which there is as small as possible an energy offset between the LE and D^+^A^−^ states^18–25^. In these instances, it can be anticipated that a strong coupling occurs between the LE and D^+^A^−^ states; such a strong coupling has potentially a significant impact on the low-energy absorption of the D/A system and, consequently, on the charge-separation efficiency as well as on the radiative and non-radiative transitions. Therefore, especially in the case of such systems that form the basis of a next generation of efficient OSCs, it is critical to rely on a robust methodology that goes beyond the conventional two-state Mulliken–Hush approach.

A number of earlier investigations on D/A small-molecule complexes or D-A isolated molecules have employed a three-state model that includes the G, D^+^A^−^, and LE states to investigate the electronic-structure properties in the framework of the perturbation theory^26,27^. Based on these early works, the impact of the LE-D^+^A^−^ electronic coupling on the intensity of the CT absorption in polymer/fullerene systems has been discussed in terms of intensity borrowing, in the context of the weak electronic coupling limit^28^. Here, we go beyond the perturbation theory and develop a three-state dynamic vibronic model that incorporates the G, D^+^A^−^, and LE states in order to describe reliably the nature of the CT absorptions in D/A active layers. Thus, the electronic couplings and electron-vibrational couplings are both treated non-perturbatively and the vibrational modes are treated quantum mechanically. Our objectives in the present work are: (i) to provide a comprehensive description of the nature of the CT absorptions in D/A complexes; (ii) to bring caution to the use of the semi-classical two-state Mulliken–Hush models as they can lead to serious misinterpretations; and (iii) to describe a first application of our methodology to the PBTTT/PCBM and PIPCP/PCBM systems that have recently attracted a great deal of attention^18,29–34^.

## Results

### Introduction to theoretical models

**Fig. 1:**
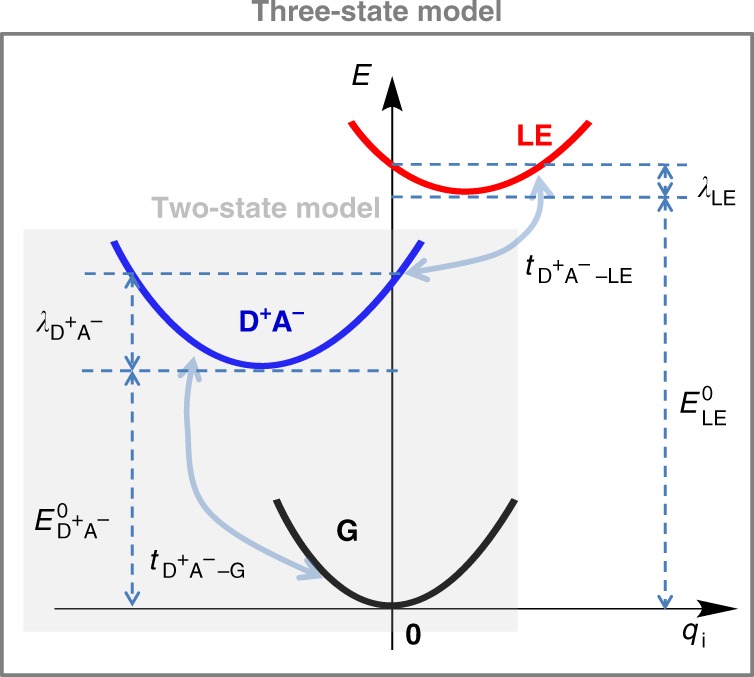
Schematic diagram of the potential energy surfaces. Schematic diagram of the potential energy surfaces for the G (black), D^+^A^−^ (blue), and LE (red) diabatic states. $E_{{\mathrm{LE}}/{\mathrm{D}}^ + {\mathrm{A}}^ - }^0$ denotes the relaxed excitation energy of the LE/D^+^A^−^ state (i.e., the excitation energy at the equilibrium geometry of the D^+^A^−^/LE state, relative to the energy at the equilibrium geometry of the G state); $\lambda _{{\mathrm{LE}}/{\mathrm{D}}^ + {\mathrm{A}}^ - }$, the relaxation energy of the LE/D^+^A^−^ state; and $t_{{\mathrm{D}}^ + {\mathrm{A}}^ - - {\mathrm{G}}}$ and $t_{{\mathrm{D}}^ + {\mathrm{A}}^ - - {\mathrm{LE}}}$, the electronic couplings of the D^+^A^−^ state with the G state and LE state, respectively

According to the semi-classical two-state Mulliken–Hush model, the CT absorption has a Gaussian shape with the following characteristics^17^:

1 $$A(E)/E = {{A}}_{{\mathrm{max}}}{\mathrm{exp}}\left( { - \frac{{(E_{{\mathrm{D}}^ + {\mathrm{A}}^ - }^0 + \lambda _{{\mathrm{D}}^ + {\mathrm{A}}^ - } - E)^2}}{{4k_{\mathrm{B}}T\lambda _{{\mathrm{D}}^ + {\mathrm{A}}^ - }}}} \right)$$

2 $$E_{{\mathrm{max}}}^{{\mathrm{CT}}} = E_{{\mathrm{D}}^ + {\mathrm{A}}^ - }^0 + \lambda _{{\mathrm{D}}^ + {\mathrm{A}}^ - }$$

3 $$\left( {{\mathrm{\Delta }}E_{1/2}^{{\mathrm{CT}}}} \right)^2 = 16{\mathrm{ln}}2k_{\mathrm{B}}T\lambda _{{\mathrm{D}}^ + {\mathrm{A}}^ - }$$

where *A*(*E*) is the optical absorption intensity (directly proportional to the extinction coefficient) per donor/acceptor pair; *E*, the photon energy; $\lambda _{{\mathrm{D}}^ + {\mathrm{A}}^ - }$, the reorganization energy related to electron transfer between the ground (G) state and the D^+^A^−^ state; $E_{{\mathrm{D}}^ + {\mathrm{A}}^ - }^0$, the relaxed D^+^A^−^-state excitation energy (Fig. 1); $E_{{\mathrm{max}}}^{{\mathrm{CT}}}$, the energy at the maximum of the CT absorption; ${\mathrm{\Delta }}E_{1/2}^{{\mathrm{CT}}}$, the full width at half maximum of the CT absorption. *k*_B_, the Boltzmann constant; and *T*, the temperature. Here, the electronic coupling, $t_{{\mathrm{D}}^ + {\mathrm{A}}^ - - {\mathrm{G}}}$, between the G and D^+^A^−^ states is related to the transition dipole moment *μ*_CT_ of the CT absorption band^35^:

4 $$t_{{\mathrm{D}}^ + {\mathrm{A}}^ - - {\mathrm{G}}} = \frac{{\mu_{{\mathrm{CT}}}}}{{e\left| {{\mathop{{\mathbf{R}}}\limits^{\rightarrow}}_{{\mathrm{ET}}}} \right|}}E_{{\mathrm{max}}}^{{\mathrm{CT}}}$$

where $| {{\mathop{{\mathbf{R}}}\limits^{\rightarrow}}_{{\mathrm{ET}}}} |$ denotes the diabatic electron-transfer (ET) distance between donor and acceptor and *e*, the electron charge. From an analysis of the CT absorption band, it is in principle possible to estimate $E_{{\mathrm{D}}^ + {\mathrm{A}}^ - }^0$ and both the electronic coupling and reorganization energy.

The three-state model is obtained by considering in addition to the G and D^+^A^−^ states a local-excitation (LE) state. The related potential energies are shown in Fig. 1. A key element of this model is that now the D^+^A^−^ state, in addition to being coupled to the ground state, is also coupled to the LE state by an electronic coupling $t_{{\mathrm{D}}^ + {\mathrm{A}}^ - - {\mathrm{LE}}}$. The related dynamic vibronic Hamiltonian and its solutions are discussed in the “Methods” section. Prior to discussing the application of the three-state approach to actual systems, such as the PBTTT/PCBM and PIPCP/PCBM active layers, it is most useful to set the stage by considering first some general model cases.

### Model cases

It is informative to consider two model cases that are related to the main geometric configurations (edge-on and face-on packing modes) appearing at the D/A interfaces: one where the axis corresponding to the electron transfer from D to A is parallel (*θ* = 0° or 180° ${\mathop{{\mathbf{R}}}\limits^{\rightarrow}}_{{\mathrm{ET}}}//\overrightarrow {\mathbf{\mu }} _{\mathrm{l}}$) or perpendicular (*θ* = 90° ${\mathop{{\mathbf{R}}}\limits^{\rightarrow}}_{{\mathrm{ET}}} \bot \overrightarrow {\mathbf{\mu }} _{\mathrm{l}}$) to the transition dipole of the LE state, here assumed to occur on the donor (e.g., in the context of pentacene/C_60_^36,37^, the ${\mathop{{\mathbf{R}}}\limits^{\rightarrow}}_{{\mathrm{ET}}}//\overrightarrow {\mathbf{\mu }} _{\mathrm{l}}$ and ${\mathop{{\mathbf{R}}}\limits^{\rightarrow}}_{{\mathrm{ET}}} \bot \overrightarrow {\mathbf{\mu }} _{\mathrm{l}}$ cases correspond to edge-on and face-on configurations, respectively); this is illustrated in Supplementary Fig. 6.

It is well-established that the details of the donor/acceptor interfacial molecular packings greatly impact the electronic structure of the CT states^15,36–38^. The polarized absorption spectra of donor/acceptor material systems are exploited experimentally to probe the nature of the CT states^28,39^, with different molecular packings showing different optical absorptions^40^. Thus, it is informative to gain a fundamental understanding of the absorption characteristics in the two limit configurations (edge-on and face-on), since the orientations of the D/A complexes in actual OSC active layers are expected to be combinations of these limit configurations. Note that, in what follows, we denote by “LE”’ the local-excitation absorbing state intrinsic to (i.e., fully localized on), say, the donor component, and by “D^+^A^−^” the pure charge-transfer state corresponding to a full electron transfer from D to A; the coupled states are denoted by “S_1_” and “CT”.

We consider three values for the offset, ${\mathrm{\Delta }}E_{{\mathrm{LE}} - {\mathrm{D}}^ + {\mathrm{A}}^ - }$, between the relaxed excitation energies ($E_{{\mathrm{LE}}}^0$ and $E_{{\mathrm{D}}^ + {\mathrm{A}}^ - }^0$, see Fig. 1) of the LE and D^+^A^−^ states: (a) 1.1 eV (8871 cm^−1^, a large value, representative, e.g., of pentacene/C_60_^36,39^ or PBTTT/PCBM^32^); (b) 0.4 eV (3226 cm^−1^, a moderate value, representing, e.g., the PBDTTPD/PCBM blend^41^); and (c) 0.2 eV (1613 cm^−1^, a small value, relevant, e.g., for PIPCP/PCBM^29^ or PNOz4T/PCBM^22^). Without any loss of generality (and in order to prevent prohibitive computational costs), we assume that the vertical excitation energy of the diabatic D^+^A^−^ state is 1.0 eV (8065 cm^−1^); although the absolute value of the D^+^A^−^-state energy chosen here is small, this choice does not affect the conclusions derived from the three-state approach since, it is the energy offset ${\mathrm{\Delta }}E_{{\mathrm{LE}} - {\mathrm{D}}^ + {\mathrm{A}}^ - }$ that mainly impacts the shapes and intensities of the whole absorption spectrum. Our vibronic model includes two effective vibrational modes^42^: a high-frequency (HF) vibration mode (ℏ*ω*_HF_ = 1200 cm^−1^), typical of a carbon–carbon bond stretch^43,44^, and a low-frequency (LF) vibration mode (ℏ*ω*_LF_ = 100 cm^−1^), which represents rotations between intramolecular fragments as well as intermolecular motions^45,46^ (with these vibrational modes, the D^+^A^−^-state relaxed excitation energy is *ca*. 0.71 eV (5713 cm^−1^)). The values of electronic couplings and electron-vibration couplings are chosen in such a way as to match commonly used literature values^36,45,47,48^; they are summarized in Table 1 (for detailed discussion, see also Supplementary Discussion 1). We consider a representative, small value of the D^+^A^−^-G electronic coupling (30 cm^−1^) while the D^+^A^−^-LE electronic coupling is varied within the range from 0 to 300 cm^−1^ in order to easily assess the role of the D^+^A^−^-LE electronic coupling in the absorption spectra.

**Table 1:** Parameters considered in the three-state vibronic model study

Parameter	Value
Energy of the high-frequency (HF) vibrational normal mode, *ħω*_HF_	1200 cm^−1^
Vibronic coupling constant corresponding to the LE state for the HF vibrational mode, $g_{{\mathrm{HF}}}^{{\mathrm{LE}}}$	0.7
Vibronic coupling constant corresponding to the D^+^A^−^ state for the HF vibrational mode, $g_{{\mathrm{HF}}}^{{\mathrm{D}}^ + {\mathrm{A}}^ - }$	0.7
Energy of the low-frequency (LF) vibrational normal mode, *ħω*_LF_	100 cm^−1^
Vibronic coupling constant corresponding to the LE state for the LF vibrational mode, $g_{{\mathrm{LF}}}^{{\mathrm{LE}}}$	2.0
Vibronic coupling constant corresponding to the D^+^A^−^ state for the LF vibrational mode, $g_{{\mathrm{LF}}}^{{\mathrm{D}}^ + {\mathrm{A}}^ - }$	4.2
Transition dipole of the LE state localized on the donor,$\left|\overrightarrow {\mathbf{\mu }} _{\mathrm{l}}\right|$	10 D
Diabatic electron-transfer (ET) distance between donor and acceptor, $\left|{\mathop{{\bf{R}}}\limits^{\rightarrow}}_{{\rm{ET}}}\right|$	8 Å
D^+^A^−^-G electronic coupling, $t_{{\mathrm{D}}^ + {\mathrm{A}}^ - - {\mathrm{G}}}$	30 cm^−1^
D^+^A^−^-LE electronic coupling, $t_{{\mathrm{D}}^ + {\mathrm{A}}^ - - {\mathrm{LE}}}$	0–300 cm^−1^

**Fig. 2:**
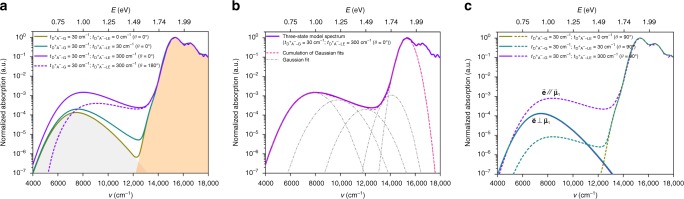
Absorption spectra simulated via the three-state vibronic approach. **a** Absorption spectra simulated via the three-state vibronic approach for the ${\mathop{{\mathbf{R}}}\limits^{\rightarrow}}_{{\mathrm{ET}}}//\overrightarrow {\mathbf{\mu }} _{\mathrm{l}}$ configuration in the case where ${\mathrm{\Delta }}E_{{\mathrm{LE}} - {\mathrm{D}}^ + {\mathrm{A}}^ - } = 1.1\,{\mathrm{eV}}$ (8871 cm^−1^); the photon polarization is taken parallel to the *X* axis (*γ* = 0°), see Supplementary Fig. 6. *E* is the photon energy; *v*, the wavenumber. The regions filled in gray and orange correspond to the D^+^A^−^ and LE absorption bands, respectively, in the absence of any D^+^A^−^-LE coupling $\left( {t_{{\mathrm{D}}^ + {\mathrm{A}}^ - - {\mathrm{LE}}} = 0\,{\mathrm{cm}}^{ - 1}} \right)$. The solid and dashed lines correspond to the simulated absorption spectra in the cases where *θ* = 0° and 180° (see Supplementary Fig. 6), respectively. **b** Decomposition into multiple Gaussian functions of the whole CT absorption band simulated via the three-state vibronic approach in the case where ${\mathrm{\Delta }}E_{{\mathrm{LE}} - {\mathrm{D}}^ + {\mathrm{A}}^ - } = 1.1\,{\mathrm{eV}}$ (8871 cm^−1^), $t_{{\mathrm{D}}^ + {\mathrm{A}}^ - - {\mathrm{G}}} = 30\,{\mathrm{cm}}^{ - 1}$, and $t_{{\mathrm{D}}^ + {\mathrm{A}}^ - - {\mathrm{LE}}} = 300\,{\mathrm{cm}}^{ - 1}$. **c** Absorption spectra simulated via the three-state vibronic approach for the ${\mathop{{\mathbf{R}}}\limits^{\rightarrow}}_{{\mathrm{ET}}} \bot \overrightarrow {\mathbf{\mu }} _{\mathrm{l}}$ configuration in the case where ${\mathrm{\Delta }}E_{{\mathrm{LE}} - {\mathrm{D}}^ + {\mathrm{A}}^ - } = 1.1\,{\mathrm{eV}}$ (8871 cm^−1^); the solid and dashed lines correspond to the simulated absorption spectra in the cases where $\overrightarrow {\mathbf{e}} \bot \overrightarrow {\mathbf{\mu }} _{\mathrm{l}}$ and $\overrightarrow {\mathbf{e}} //\overrightarrow {\mathbf{\mu }} _{\mathrm{l}}$ (see Supplementary Fig. 6), respectively

We start with a discussion of the results in the case where ${\mathrm{\Delta }}E_{{\mathrm{LE}} - {\mathrm{D}}^ + {\mathrm{A}}^ - } = 1.1\,{\mathrm{eV}}$ (8871 cm^−1^) for the configuration where the electron-transfer axis is parallel (*θ* = 0°) to the transition dipole of the LE state, i.e., ${\mathop{{\mathbf{R}}}\limits^{\rightarrow}}_{{\mathrm{ET}}}//\overrightarrow {\mathbf{\mu }} _{\mathrm{l}}$, see Fig. 2a. In this instance, in qualitative agreement with the experimental data^7^ (e.g., for the tetracene/C_60_ bilayer system in an edge-on configuration^49^), a low-energy absorption shoulder, with an intensity some 10^2^–10^4^ times weaker than that of the pure donor absorption, can be clearly distinguished. When the D^+^A^−^ state is considered not to be coupled with the LE state (i.e., $t_{{\mathrm{D}}^ + {\mathrm{A}}^ - - {\mathrm{LE}}} = 0\,{\mathrm{cm}}^{ - 1}$), the absorption spectrum of the D/A systems corresponds to a simple superposition of the D^+^A^−^ and LE absorption bands; in this instance, as should be obviously the case, the transition dipole moment (*μ*_CT_) for the CT absorption derived from our vibronic solutions (0.15 D for $t_{{\mathrm{D}}^ + {\mathrm{A}}^ - - {\mathrm{G}}} = 30\,{\mathrm{cm}}^{ - 1}$) matches the value estimated from the semi-classical two-state Mulliken–Hush model (0.14 D); we note, however, that the vibronic model predicts a slightly red-shifted, asymmetric, and wider absorption band (the energy at the maximum of the CT absorption $E_{{\mathrm{max}}}^{{\mathrm{CT}}} = 7460\,{\mathrm{cm}}^{ - 1}(0.92{\mathrm{eV}})$ and the full width at half maximum of the CT absorption ${\mathrm{\Delta }}E_{1/2}^{{\mathrm{CT}}} = 2478\,{\mathrm{cm}}^{ - 1}$ (0.31 eV)) than the semi-classical Mulliken–Hush model ($E_{{\mathrm{max}}}^{{\mathrm{CT}}} = 8066\,{\mathrm{cm}}^{ - 1}$ (1.00 eV) and ${\mathrm{\Delta }}E_{1/2}^{{\mathrm{CT}}} = 2284\,{\mathrm{cm}}^{ - 1}$ (0.28 eV)), which can be attributed to the quantum treatment of the vibrations in our approach. If the Marcus–Levich–Jortner (MLJ) approach^50^ that also treats the high-frequency vibrations quantum mechanically is used to simulate the CT absorption band, a better agreement with our present results is indeed obtained (see Supplementary Fig. 1 and Supplementary Discussion 2), which is consistent with the results of Köhler et al.^51^. Thus, observing clearly a distinct, symmetric low-energy shoulder is an indication that the D^+^A^−^-LE coupling is vanishingly small (then, a two-state model can be used, however, such a situation is not conducive to efficient exciton dissociation).

As seen from Fig. 2a, introducing the coupling between the D^+^A^−^ state and the LE state has a marked effect on both the shape and intensity of the resulting CT absorption band. There are two contributions to the CT absorption band (see Eq. 6 in the “Methods” section): the first is related to the pure D^+^A^−^ state itself while the second is due to the hybridization between the intrinsic LE and D^+^A^−^ states (corresponding to an intensity borrowing effect). Depending on the relative orientations between the dipole moment of the D^+^A^−^state $\left( {e{\mathop{{\mathbf{R}}}\limits^{\rightarrow}}_{{\mathrm{ET}}}} \right)$ and the transition dipole moment of the LE state $\left( {\overrightarrow {\mathbf{\mu }} _{\mathrm{l}}} \right)$, here assumed to occur on the donor, these contributions can act either in a constructive or in a destructive manner^27^. In the case where *θ* = 0°, these contributions are constructive. As a result, in comparison with the CT absorption band derived when $t_{{\mathrm{D}}^ + {\mathrm{A}}^ - - {\mathrm{LE}}}$ is set to 0 cm^−1^, an increase in $t_{{\mathrm{D}}^ + {\mathrm{A}}^ - - {\mathrm{LE}}}$ coupling leads to broader, more intense, slightly blue-shifted, and more asymmetric CT absorption bands (see Fig. 2a and Table 2a). Importantly, an increase in $t_{{\mathrm{D}}^ + {\mathrm{A}}^ - - {\mathrm{LE}}}$ coupling from 0 to 300 cm^−1^ (typical of OSC systems^52,53^) results in an increase in absorption intensity by one order of magnitude.

**Table 2:** **a** $E_{{\mathrm{max}}}^{{\mathrm{CT}}}$, ${\mathrm{\Delta }}E_{1/2}^{{\mathrm{CT}}}$, and *μ*_CT_ of the CT absorption band derived from the three-state calculations when ${\mathrm{\Delta }}E_{{\mathrm{LE}} - {\mathrm{D}}^ + {\mathrm{A}}^ - } = 1.1\,{\mathrm{eV}}$ (8871 cm^−1^) and $t_{{\mathrm{D}}^ + {\mathrm{A}}^ - - G} = 30\,{\mathrm{cm}}^{ - 1}$

$t_{{\mathrm{D}}^ + {\mathrm{A}}^ - - {\mathrm{LE}}}\left( {{\mathrm{cm}}^{ - 1}} \right)$	$E_{{\mathrm{max}}}^{{\mathrm{CT}}}\left( {{\mathrm{cm}}^{ - 1}} \right)$	${\mathrm{\Delta }}E_{1/2}^{{\mathrm{CT}}}\left( {{\mathrm{cm}}^{ - 1}} \right)$	*μ*_CT _(D)
**a**			
0	7460	2478	0.15
30	7590	2646	0.19
300	8020	3265	0.56
**b**			
$t_{{\mathrm{D}}^ + {\mathrm{A}}^ - - {\mathrm{LE}}}$ (cm^−1^)	$\lambda _{{\mathrm{D}}^ + {\mathrm{A}}^ - }$ (cm^−1^)	$E_{{\mathrm{D}}^ + {\mathrm{A}}^ - }^0$ (cm^−1^)	$t_{{\mathrm{D}}^ + {\mathrm{A}}^ - - {\mathrm{G}}}$ (cm^−1^)
0	2768 (1871)	4692 (5429)	29
30	3156 (1924)	4434 (5476)	38
300	4806 (2185)	3214 (5646)	117
Input parameters	2352	5713	30

Here, the $E_{{\mathrm{max}}}^{{\mathrm{CT}}}$ and ${\mathrm{\Delta }}E_{1/2}^{{\mathrm{CT}}}$ value are directly measured from the whole CT absorption band; the *μ*_CT_ value is obtained through integrating this whole band. **b** $\lambda _{{\mathrm{D}}^ + {\mathrm{A}}^ - }$, $E_{{\mathrm{D}}^ + {\mathrm{A}}^ - }^0$, and $t_{{\mathrm{D}}^ + {\mathrm{A}}^ - - {\mathrm{G}}}$ values obtained by using the Mulliken–Hush Equations 2 to 4 in the analysis of the whole CT absorption bands derived from the three-state calculations when ${\mathrm{\Delta }}E_{{\mathrm{LE}} - {\mathrm{D}}^ + {\mathrm{A}}^ - } = 1.1{\mathrm{eV}}\,(8871\,{\mathrm{cm}}^{ - 1})$ and $t_{{\mathrm{D}}^ + {\mathrm{A}}^ - - {\mathrm{G}}} = 30\,{\mathrm{cm}}^{ - 1}$; the values in parentheses are obtained by a Gaussian fit (see Fig. 2b, and Supplementary Fig. 3) via Eq. (1) to the low-energy tails of the CT absorption bands derived from the three-state calculations. The “input parameters” row denotes the parameters used in our three-state calculations

It is instructive at this point to address how the CT absorption bands derived on the basis of an explicit consideration of the couplings among the LE, D^+^A^−^, and G states would be described by the semi-classical two-state model, which we recall is the model that has been applied to date in order to characterize the CT absorption bands. There are two procedures commonly used to interpret the CT absorption bands:

(a) The Mulliken–Hush Eqs (2) and (3) are employed analytically to extract the relaxed excitation energies and reorganization energies of the CT states, given that the energies at the maximum of the CT absorption and the full widths at half maximum of the CT absorption can be directly measured. Through integrating the whole CT absorption bands, the transition dipole moments (*μ*_CT_) can be obtained, and the electronic couplings $t_{{\mathrm{D}}^ + {\mathrm{A}}^ - - {\mathrm{G}}}$ with the ground states can then be derived via Eq. (4). This procedure is usually followed in the research community focusing on intervalence-transfer absorption bands (e.g., mixed-valence systems)^17,54^. Here, taking as example the absorption spectrum simulated via our three-state model in the case where ${\mathrm{\Delta }}E_{{\mathrm{LE}} - {\mathrm{D}}^ + {\mathrm{A}}^ - } = 1.1\,{\mathrm{eV}}\,(8871\,{\mathrm{cm}}^{ - 1})$ and $t_{{\mathrm{D}}^ + {\mathrm{A}}^ - - {\mathrm{G}}} = 30\,{\mathrm{cm}}^{ - 1}$, we used this procedure to analyze the whole CT absorption band, and the relevant microscopic parameters $E_{{\mathrm{D}}^ + {\mathrm{A}}^ - }^0$, $\lambda _{{\mathrm{D}}^ + {\mathrm{A}}^ - }$ and $t_{{\mathrm{D}}^ + {\mathrm{A}}^ - - {\mathrm{G}}}$ derived in this way are given in Table 2b. The comparison between the Mulliken–Hush analysis results and the input parameters in our three-state model (see Table 2b) reveals that, as the $t_{{\mathrm{D}}^ + {\mathrm{A}}^ - - {\mathrm{LE}}}$ coupling increases, the traditional two-state Mulliken–Hush model increasingly: (a) overestimates the electronic coupling between the D^+^A^−^ state and the ground state since the absorption intensity (or transition dipole moment) borrowed from the LE state is incorrectly attributed to the D^+^A^−^ state itself; (b) overestimates the relaxation energy in the D^+^A^−^ state; and (c) importantly underestimates the D^+^A^−^-state energy, $E_{{\mathrm{D}}^ + {\mathrm{A}}^ - }^0$. In order to prevent any confusion, we note that for all ${\mathrm{\Delta }}E_{{\mathrm{LE}} - {\mathrm{D}}^ + {\mathrm{A}}^ - }$ and $t_{{\mathrm{D}}^ + {\mathrm{A}}^ - - {\mathrm{G}}}$ values considered in this work, the energies of the relaxed adiabatic CT states nearly coincide with the energies, $E_{{\mathrm{D}}^ + {\mathrm{A}}^ - }^0$, of the diabatic states (see also Supplementary Discussion 3).

(b) In the OPV community, the usual procedure is to use a Gaussian function to fit the low-energy absorption tails, in order to extract the electronic-structure parameters of the CT states^11^. For example, for the absorption spectrum simulated via our three-state model in the case where ${\mathrm{\Delta }}E_{{\mathrm{LE}} - {\mathrm{D}}^ + {\mathrm{A}}^ - } = 1.1\,{\mathrm{eV}}\,(8871\,{\mathrm{cm}}^{ - 1})$, $t_{{\mathrm{D}}^ + {\mathrm{A}}^ - - {\mathrm{G}}} = 30\,{\mathrm{cm}}^{ - 1}$, and $t_{{\mathrm{D}}^ + {\mathrm{A}}^ - - {\mathrm{LE}}} = 300\,{\mathrm{cm}}^{ - 1}$, when this procedure is used to fit the low-energy absorption tail (see Fig. 2b and Table 2b), the fitted $E_{{\mathrm{D}}^ + {\mathrm{A}}^ - }^0$ value (5646 cm^−1^ (0.70 eV)) is close to the input value in our three-state model (5713 cm^−1^ (0.71 eV)); on the other hand, the fitted $\lambda _{{\mathrm{D}}^ + {\mathrm{A}}^ - }$ (2185 cm^−1^ (0.27 eV)) is smaller than our input value (2352 cm^−1^ (0.29 eV)), which comes from the fact that the quantum effect of high-frequency vibration is neglected in the semi-classical two-state model. When a series of Gaussian functions is used to reproduce the whole CT absorption band (see Fig. 2b), the generated higher-energy absorption peaks are generally assigned to higher-energy CT states (i.e., CT_2_, CT_3_, …, CT_n_), as done in the recent work of Belova et al.^40^ However, according to the three-state approach, these higher-energy absorptions correspond in fact to hot hybrid (D^+^A^−^-LE) vibronic states, which are induced by D^+^A^−^-LE coupling. Thus, our results bring to light another explanation for the higher-energy absorptions observed experimentally^15,40^.

In the case where *θ* = 180°, the contributions of the D^+^A^−^ and LE states to the CT absorptions act in a destructive fashion. As a result, the D^+^A^−^ and LE-state contributions cancel each other, especially in the low-energy tail (see also Fig. 2a). As the $t_{{\mathrm{D}}^ + {\mathrm{A}}^ - - {\mathrm{LE}}}$ coupling increases, the vibronic states gain more weight from the LE state (the closer these states to the LE state, the larger the weights). Consequently, the CT absorption band shows a blue-shift.

We now turn to a discussion of the ${\mathop{{\mathbf{R}}}\limits^{\rightarrow}}_{{\mathrm{ET}}} \bot \overrightarrow {\mathbf{\mu }} _{\mathrm{l}}$ configuration. Given that in this case $e{\mathop{{\mathbf{R}}}\limits^{\rightarrow}}_{{\mathrm{ET}}}$ and $\overrightarrow {\mathbf{\mu }} _{\mathrm{l}}$ are perpendicular (see Supplementary Fig. 6), the respective contributions of the D^+^A^−^ and LE states to the CT absorptions can be decoupled as a function of photon polarization. When photon polarization is parallel to $e{\mathop{{\mathbf{R}}}\limits^{\rightarrow}}_{{\mathrm{ET}}}$, i.e., to the electron-transfer axis, only the component of the vibronic wavefunctions (see Eq. 6) proportional to $\left| {{\mathrm{\Phi }}_{{\mathrm{D}}^ + {\mathrm{A}}^ - }} \right\rangle$ contributes to the transition dipole moment; as a consequence, the CT absorption band resembles that obtained in the framework of the two-state model (see Fig. 2c). On the other hand, when light polarization is parallel to $\overrightarrow {\mathbf{\mu }} _{\mathrm{l}}$, the CT absorption derives entirely from the coupling between the D^+^A^−^ and LE states (see Eq. 6); it is interesting to realize that such CT absorptions are not dependent on the coupling between the D^+^A^−^ and ground states. As the $t_{{\mathrm{D}}^ + {\mathrm{A}}^ - - {\mathrm{LE}}}$ coupling increases, the contribution of the LE state to the low-energy vibronic states also increases, which makes the CT absorption band more intense and more extended toward lower energies (see Fig. 2c). Thus, an important implication of these results is that a prominent low-energy feature in the absorption spectra of the D/A blends can appear even when the ground state and the D^+^A^−^ states are not electronically coupled $\left( {t_{{\mathrm{D}}^ + {\mathrm{A}}^ - - {\mathrm{G}}} = 0\,{\mathrm{cm}}^{ - 1}} \right)$. Thus, in such a case, the application of the two-state model into the analysis/fitting of these CT absorption bands would lead to erroneous CT-state electronic-structure characteristics.

**Fig. 3:**
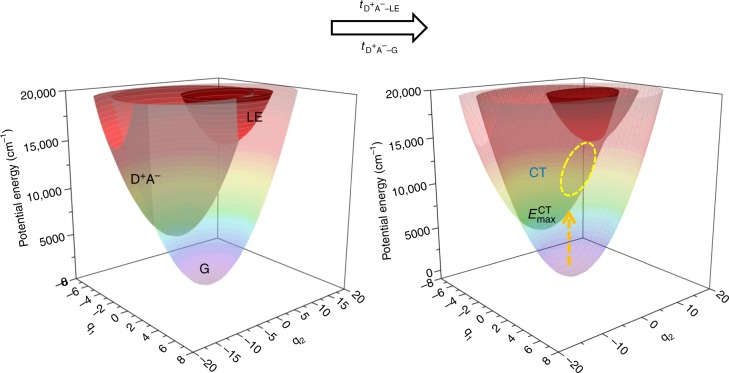
Diagram of the potential energy surfaces. Left: diagram of the potential energy surfaces for the G, D^+^A^−^ and LE intrinsic (diabatic) states when ${\mathrm{\Delta }}E_{{\mathrm{LE}} - {\mathrm{D}}^ + {\mathrm{A}}^ - } = 1.1{\mathrm{eV}}\,(8871\,{\mathrm{cm}}^{ - 1})$; right: diagram of the potential energy surfaces for the adiabatic states when ${\mathrm{\Delta }}E_{{\mathrm{LE}} - {\mathrm{D}}^ + {\mathrm{A}}^ - } = 1.1\,{\mathrm{eV}}\,(8871\,{\mathrm{cm}}^{ - 1})$, $t_{{\mathrm{D}}^ + {\mathrm{A}}^ - - {\mathrm{G}}} = 30\,{\mathrm{cm}}^{ - 1}$, and $t_{{\mathrm{D}}^ + {\mathrm{A}}^ - - {\mathrm{LE}}} = 300\,{\mathrm{cm}}^{ - 1}$. Here, $E_{{\mathrm{max}}}^{{\mathrm{CT}}}$ is the energy of the maximum CT absorption derived in the semi-classical two-state model; the region circled by the yellow dashed line represents the ‘hot’ CT vibronic states

A general consequence of the coupling between the D^+^A^−^ and LE states is that the “hot” CT vibronic states (see the region circled by the yellow dashed line in the diagram of the potential energy surfaces shown in Fig. 3) represent the hybrid D^+^A^−^-LE states (for details, see Supplementary Discussion 3) and are therefore characterized by a significant transition dipole moment (see Table 2a). They can thus be efficiently directly accessed via optical excitation and could open a direct pathway to dissociation into free charge carriers, as soon as their energies are located above charge-separated states, or below them but within thermal excitation energy^6^. We also note that these hybrid states, especially in the case where ${\mathrm{\Delta }}E_{{\mathrm{LE}} - {\mathrm{D}}^ + {\mathrm{A}}^ - }$ is small, could contribute to increase the radiative recombination rate^27^, thus to enhance the radiative efficiency, which would eventually lead to a smaller non-radiative voltage loss^20,23^. In addition, an increase in radiative recombination rate can also mean an increase in photocurrent.

**Fig. 4:**
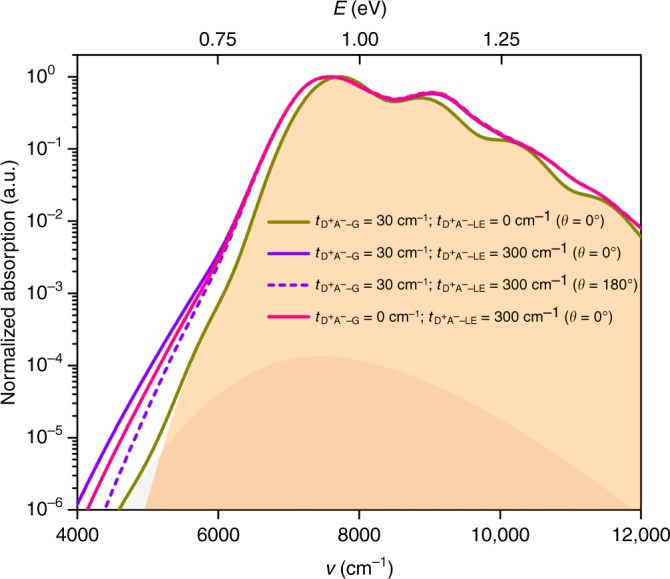
Absorption spectra simulated via the three-state vibronic model. Absorption spectra simulated via the three-state vibronic model for the ${\mathop{{\mathbf{R}}}\limits^{\rightarrow}}_{{\mathrm{ET}}}//\overrightarrow {\mathbf{\mu }} _{\mathrm{l}}$ configuration, when the electric field of the light is parallel to the transition dipole of the LE state (*γ* = 0°), in the case where the ${\mathrm{\Delta }}E_{{\mathrm{LE}} - {\mathrm{D}}^ + {\mathrm{A}}^ - } = 0.2\,{\mathrm{eV}}$ (1613 cm^−1^). The regions filled in gray and orange correspond to the D^+^A^−^ and LE absorption bands, respectively, as derived from a two-state vibronic model (*i*.*e*., in the absence of any $t_{{\mathrm{D}}^ + {\mathrm{A}}^ - - {\mathrm{LE}}}$ coupling). The solid and dashed lines correspond to the simulated absorption spectra in the cases where *θ* = 0° and 180° (see Supplementary Fig. 6), respectively

The results obtained for the case where the offset, ${\mathrm{\Delta }}E_{{\mathrm{LE}} - {\mathrm{D}}^ + {\mathrm{A}}^ - }$, between the relaxed excitation energies of the LE and D^+^A^−^ states comes down to 0.4 eV (see Supplementary Fig. 4) turn out to very much resemble those discussed above for ${\mathrm{\Delta }}E_{{\mathrm{LE}} - {\mathrm{D}}^ + {\mathrm{A}}^ - } = 1.1\,{\mathrm{eV}}$. Thus, they are not further discussed here and we focus next on the instance where ${\mathrm{\Delta }}E_{{\mathrm{LE}} - {\mathrm{D}}^ + {\mathrm{A}}^ - }$ becomes smaller than 0.4 eV, in which case significant differences start to appear. Such systems are in fact currently drawing great interest in order to potentially reduce the voltage loss^13,18–20,22,55,56^. The results obtained for the ${\mathop{{\mathbf{R}}}\limits^{\rightarrow}}_{{\mathrm{ET}}}//\overrightarrow {\mathbf{\mu }} _{\mathrm{l}}$ configuration in the ${\mathrm{\Delta }}E_{{\mathrm{LE}} - {\mathrm{D}}^ + {\mathrm{A}}^ - } = 0.2\,{\mathrm{eV}}$ (1613 cm^−1^) case are shown in Fig. 4 (see also Supplementary Fig. 5). When the D^+^A^−^ state is not coupled with the LE state, the CT absorption for small $t_{{\mathrm{D}}^ + {\mathrm{A}}^ - - {\mathrm{G}}}$ couplings is completely buried within the absorption of the neat donor; it is only for larger $t_{{\mathrm{D}}^ + {\mathrm{A}}^ - - {\mathrm{G}}}$ values (e.g., 300 cm^−1^) that a slight shoulder appears in the low-energy region of the absorption spectrum (see Supplementary Fig. 5). Importantly, the inclusion of the $t_{{\mathrm{D}}^ + {\mathrm{A}}^ - - {\mathrm{LE}}}$ coupling does not lead to any prominent low-energy absorption shoulder. Instead, such coupling is observed to lead to a red-shift of the whole low-energy edge of the absorption spectrum. In the case where $t_{{\mathrm{D}}^ + {\mathrm{A}}^ - - {\mathrm{G}}} = 30\,{\mathrm{cm}}^{ - 1}$ and $t_{{\mathrm{D}}^ + {\mathrm{A}}^ - - {\mathrm{LE}}} = 300\,{\mathrm{cm}}^{ - 1}$, the low-energy part of the absorption spectrum, for reasons discussed earlier, slightly depends on the mutual orientations of dipole moments $\overrightarrow {\mathbf{\mu }} _{\mathrm{l}}$ and $e{\mathop{{\mathbf{R}}}\limits^{\rightarrow}}_{{\mathrm{ET}}}$; overall, the results remain very close to those obtained for $t_{{\mathrm{D}}^ + {\mathrm{A}}^ - - {\mathrm{G}}} = 0$.

### Applications of the three-state approach

**Fig. 5:**
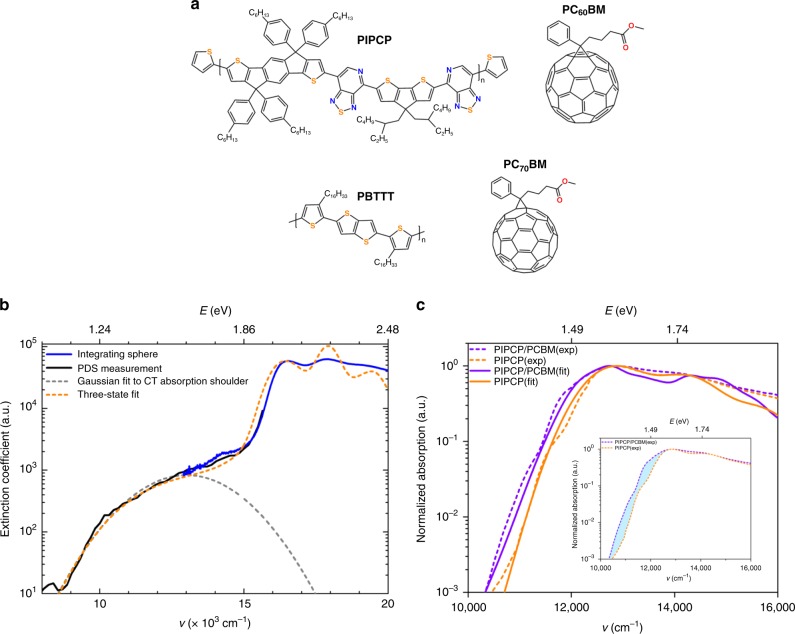
Applications of the three-state approach. **a** Chemical structures of PIPCP, PC_60_BM, PBTTT, and PC_70_BM. **b** Experimental and fitted extinction coefficient of the PBTTT/PC_70_BM blend. Since photo-thermal deflection spectroscopy (PDS) can only be used to determine the spectrum shape of CT absorption and not its absolute value, in the experiment^32^ the PDS spectrum (black line) was therefore scaled to absolute absorption units by matching the PDS spectrum in the strongly absorbing region with an integrating sphere UV-vis transmission and reflection measurement (blue line); a Gaussian fit (dashed gray line) was used to model the experimental CT absorption shoulder in the experiment^32^. Here, our three-state fit (dashed yellow line) is also employed to fit the full absorption spectrum of PBTTT/PC_70_BM. **c** Experimental (dashed lines) and fitted (solid lines) absorption spectra of pure PIPCP and the PIPCP/PC_60_BM blend. The experimental absorption spectra of the pure PIPCP polymer and the PIPCP/PC_60_BM blend were obtained *via* PDS measurements^29^. In the inset, the region filled in blue shows an additional absorption feature at the low-energy edge of the blend absorption compared with that of the pure polymer

The three-state approach is now applied to the understanding of the absorption spectra in two relevant experimental systems, i.e., the PBTTT/PCBM blend reported by Sweetnam et al.^32^ and the PIPCP/PCBM blend reported by Ran et al.^29^ (see the chemical structures in Fig. 5a). Before discussing the results, it is worthwhile to briefly describe the fitting procedure: First, we fit the LE exciton absorption band by using a two-state model (Eqs. (11)–(13); here, the two-state vibronic model includes only LE and G states in Eq. 11) to obtain the LE-state energy, $E_{{\mathrm{LE}}}^0$, and the reorganization energy, λ_LE_; then, based on these parameters, we apply the three-state model Eqs. (11)–(13) to fit the low-energy part (that contains electronic-structure information of both CT and LE states) of the absorption spectrum of the donor/acceptor complex and thus extract the parameters $E_{{\mathrm{D}}^ + {\mathrm{A}}^ - }^0$, $\lambda _{{\mathrm{D}}^ + {\mathrm{A}}^ - }$, $t_{{\mathrm{D}}^ + {\mathrm{A}}^ - - {\mathrm{G}}}$, and $t_{{\mathrm{D}}^ + {\mathrm{A}}^ - - {\mathrm{LE}}}$.

We first apply our three-state approach to the PBTTT/PCBM system, which is representative of systems with large ${\mathrm{\Delta }}E_{{\mathrm{LE}} - {\mathrm{D}}^ + {\mathrm{A}}^ - }$ values^32,33^. A low-energy shoulder is clearly observed in the experimental absorption spectra, with an intensity about 100 times weaker than that of the neat polymer absorption (see Fig. 5b). In the experimental work, the use of the two-state Gaussian fit to the low-energy absorption shoulder yields values of *ca*. 1.48 D, 13000 cm^−1^ (1.61 eV), and 3510 cm^−1^ (0.44 eV) for *μ*_CT_, $E_{{\mathrm{max}}}^{{\mathrm{CT}}}$, and ${\mathrm{\Delta }}E_{1/2}^{{\mathrm{CT}}}$, respectively^32^. Based on this Gaussian fit^32^, the relaxed D^+^A^−^-state energy $E_{{\mathrm{D}}^ + {\mathrm{A}}^ - }^0$ and the relaxation energy $\lambda _{{\mathrm{D}}^ + {\mathrm{A}}^ - }$ are extracted to be 7445 cm^−1^ (0.92 eV) and 5555 cm^- 1^ (0.69 eV), respectively, and the D^+^A^−^-G electronic coupling $t_{{\mathrm{D}}^ + {\mathrm{A}}^ - - {\mathrm{G}}}$ is evaluated via Eq. (4) to be *ca*. 573 cm^−1^ (0.07 eV). In the framework of our three-state model, a very good comparison between the experiment and fit is obtained (see Fig. 5b) when $t_{{\mathrm{D}}^ + {\mathrm{A}}^ - - {\mathrm{G}}}$, $E_{{\mathrm{D}}^ + {\mathrm{A}}^ - }^0$, $\lambda _{{\mathrm{D}}^ + {\mathrm{A}}^ - }$ and the D^+^A^−^-LE coupling $t_{{\mathrm{D}}^ + {\mathrm{A}}^ - - {\mathrm{LE}}}$ are set at 300 cm^−1^ (0.04 eV), 8400 cm^−1^ (1.04 eV), 5055 cm^−1^ (0.63 eV), and 450 cm^−1^ (0.06 eV) (see also Supplementary Table 1). In agreement with the discussion for the model cases, the application of the two-state model leads to (a) an overestimation of the electronic coupling between the D^+^A^−^ state and the ground state by *ca*. 270 cm^−1^ (0.03 eV); (b) an underestimation of the relaxed D^+^A^−^-state energy by 1000 cm^−1^ (0.12 eV); and (c) an overestimation of the relaxation energy in the D^+^A^−^ state by *ca*. 500 cm^−1^ (0.06 eV). Note also that the results of a two-state Gaussian fit specifically depend on the chosen procedure, which can sometimes lead to a significant error.

We now turn to the PIPCP/PCBM system (see the chemical structures in Fig. 5a) that has drawn significant recent attention due to its peculiar absorption spectrum and smaller apparent *V*_OC_ loss of about 0.52 eV (4193 cm^−1^)^29^. The experimental absorption spectrum in the blend is red-shifted by *ca*. 60 meV (484 cm^−1^) compared with that of the pure polymer, with no appearance of a low-energy CT absorption shoulder in either photo-thermal deflection spectroscopy (PDS) or external quantum efficiency (EQE) spectra^29^. The lack of a low-energy CT absorption shoulder in the experimental EQE spectra has also been reported in other, high-efficient systems with low-energy loss^20,55,56^. Now, we exploit the three-state vibronic model to fit the absorption spectrum of the PIPCP/PCBM blend.

The experimental high-resolution transmission electron microscopy indicates that PIPCP in the blend has a somewhat higher crystallinity than in the polymer neat film^29^. Therefore, the observed red-shift for the blend absorption can be attributed, at least in part, to an increase in the degree of conjugation (hole delocalization) of the PIPCP backbone when going from the neat polymer film to the blend structure^29^. However, even when the absorption spectrum of the blend is displaced so that its vibrational structure matches that of the neat polymer absorption, the blend still shows an additional absorption feature at the low-energy edge (see the inset figure in Fig. 5c). This feature can be well-accounted for when applying our three-state vibronic model. An appropriate fit (see Fig. 5c) to the experimental data is obtained with the following parameters (see also Supplementary Table 2): an LE-D^+^A^−^ energy offset ${\mathrm{\Delta }}E_{{\mathrm{LE}} - {\mathrm{D}}^ + {\mathrm{A}}^ - }$ of 0.2 eV (1613 cm^−1^); a $t_{{\mathrm{D}}^ + {\mathrm{A}}^ - - {\mathrm{G}}}$ coupling of 100 cm^−1^; and a $t_{{\mathrm{D}}^ + {\mathrm{A}}^ - - {\mathrm{LE}}}$ coupling of 400 cm^−1^ (note that, given the constraints related to reproducing the whole absorption band, a minor variation in the microscopic parameters lead to a less satisfactory fit). Interestingly, the D^+^A^−^-LE electronic coupling is seen to be much larger than the D^+^A^−^-G coupling; this result is in fact consistent with the electronic couplings calculated via long-range corrected density functional theory (see Supplementary Table 3). Moreover, this result gives a value for the (*E*_CT_ – *eV*_OC_) energy loss of only *ca*. 0.4 eV (3226 cm^−1^). While there remain differences between the fitted and experimental spectra, the three-state approach is able to reproduce the main characteristics of the absorption spectrum: (1) the absence of any low-energy absorption shoulder; (2) the presence of vibronic peaks in the region above 1.6 eV (12904 cm^−1^); and (3) the red-shift of the low-energy absorption edge of the blend compared with that of the pure polymer. The three-state vibronic model locates the D^+^A^−^ state about 0.2 eV (1613 cm^−1^) below the polymer LE state, which is about four times larger than the value of 50 meV (403 cm^−1^) estimated previously from the electroluminescence spectrum^29^.

We note that the rate of exciton dissociation in PIPCP/PCBM can be crudely estimated via Marcus electron-transfer theory where, following earlier investigations^37,57,58^, we assume the related reorganization energy to be *ca*. 0.3 eV (2419 cm^−1^). With ${\mathrm{\Delta }}E_{{\mathrm{LE}} - {\mathrm{D}}^ + {\mathrm{A}}^ - } = 0.2\,{\mathrm{eV}}$ (1613 cm^−1^) and $t_{{\mathrm{D}}^ + {\mathrm{A}}^ - - {\mathrm{LE}}} = 400\,{\mathrm{cm}}^{ - 1}$, the estimated rate is *ca*. 1 × 10^14^ s^−1^. The experimental data of Menke et al. have shown that, in the PIPCP/PCBM blend, exciton dissociation after photoexcitation is very fast (smaller than 100 fs)^31^, which is consistent with a reasonable 0.2 eV (1613  cm^−1^) driving force and a large D^+^A^−^-LE electronic coupling.

Finally, we would like to point out that our present three-state approach is a starting point and does not consider other factors such as static disorder or electronic delocalization due to aggregation effects, which can also impact the CT absorption spectra^51,59^. As reported by Köhler et al. ^51^, static disorder can lead to a broadening of the CT absorption spectrum. Thus, when neglecting static disorder, the CT-state reorganization energies $\lambda _{{\mathrm{D}}^ + {\mathrm{A}}^ - }$ could be somewhat overestimated since the spectral broadening that would be induced by static disorder is here fully attributed to molecular vibrations (i.e., dynamic disorder). In addition, electronic delocalization due to molecular aggregation could also lead to some low-energy absorptions^59^. Thus, our objective in future work will be to account for static disorder and electronic delocalization, although their inclusion is not expected to affect the main conclusions of the present work regarding the impact of the electronic coupling between the D^+^A^−^ and LE states.

## Discussion

We have developed a three-state vibronic model, with which we can explicitly take into account the electronic and vibronic couplings between the charge-transfer states appearing at the donor/acceptor interfaces and not only the ground state but also the strongly absorbing, local-excitation (LE) state on the donor and/or acceptor. We recall that coupling to only the ground state (two-state model) has been the hallmark of the analysis tools commonly applied to date to characterize the CT states appearing in the active layers of OSC. Here, the application of the three-state vibronic model has allowed us to clarify the impact of hybridization of the pure charge-transfer (D^+^A^−^) and LE states on the characteristics of the CT states at the D/A interfaces.

A number of important conclusions can be drawn:

(i) In the case of a large energy offset (e.g., greater than or equal to 0.4 eV) between the D^+^A^−^ and LE states, a low-energy CT-absorption shoulder is clearly apparent. However, the electronic coupling between the D^+^A^−^ and LE states makes the shape and intensity of the low-energy shoulder substantially different from those that the semi-classical two-state Mulliken–Hush model would predict. In fact, application of the semi-classical Mulliken–Hush model to analyze the whole CT absorption bands leads to an overestimation of the reorganization energy and electronic coupling with the ground state and an underestimation of the adiabatic energy of the D^+^A^−^ state. In addition, if a Gaussian fit to the low-energy CT absorption tails is performed, this procedure could provide a good fit to the adiabatic energy of the D^+^A^−^ state, but also misinterpret the higher-energy absorptions (which correspond to hot hybrid (D^+^A^−^-LE) vibronic states induced by the D^+^A^−^-LE coupling).
(ii) In comparison with the characteristics of the (two-state derived) conventional CT states, the hot CT vibronic states resulting from D^+^A^−^-LE coupling gain a more substantial transition dipole moment; these hot CT vibronic states, which are directly optically accessible, have energies that can allow them overcome, entirely or at least in part, the electron-hole Coulomb barrier and to dissociate easily into free charge carriers. This feature could thus result in improved power conversion efficiencies.
(iii) In the case of a small energy offset (e.g., less than or equal to 0.2 eV) between the D^+^A^−^ and LE states, which is representative of the next generation of highly efficient OSCs, the absorption spectra hardly show any low-energy absorption feature. Instead, the D^+^A^−^-LE coupling leads to a red-shift of the whole low-energy absorption edge, which is consistent with the experimental data reported for the PIPCP/PCBM blend. In such instances, a three-state model is mandatory in order to extract reliable information on the CT states from the absorption spectra.

Overall, our work provides a robust and comprehensive framework to understand the CT absorption spectra at donor/acceptor interfaces and also provides a pathway for a reliable extraction of the electronic-structure parameters describing the CT states from these spectra. However, more experimental data, such as temperature dependence and polarization dependence of the absorption, would be desirable to achieve these parameters. Our model can be further applied to multiple donor/acceptor complexes in order to account for the effects of electron delocalization and static disorder. Taking into account a three-state vibronic approach or its extensions is necessary to adequately characterize the voltage (or energy) loss mechanisms in efficient OSC devices and to guide the development of improved systems.

## Methods

The total Hamiltonian for a donor/acceptor molecular pair can be written as:

5 $$\hat H = \hat T_{\mathrm{N}} + \hat H_{\mathrm{e}}\,{\mathrm{and}}\,\hat H_{\mathrm{e}} = \hat T_{\mathrm{e}} + \hat V(r,q)$$

Here, $\hat T_{\mathrm{N}}$ denotes the kinetic energy of the nuclei; $\hat H_{\mathrm{e}}$ is the electronic part that includes the kinetic energy $\hat T_{\mathrm{e}}$ of the electrons and all (e.g., electron–electron, electron–nuclear, and nuclear–nuclear) interactions described by $\hat V(r,q)$; *r* and *q* are standard notations for the electronic and nuclear coordinates, respectively.

In the basis of the three electronic states $|{\mathrm{\Phi }}_{\mathrm{G}}\rangle$, $|{\mathrm{\Phi }}_{{\mathrm{LE}}}\rangle$, and $|{\mathrm{\Phi }}_{{\mathrm{D}}^ + {\mathrm{A}}^ - }\rangle$, corresponding to the G, LE, and D^+^A^−^ diabatic states, respectively, the *α*th eigenfunction $\Psi _{\mathrm{\alpha }}$ of the total Hamiltonian can be expressed as:

6 $$\Psi _{\mathrm{\alpha }} = \eta _{\mathrm{G}}^{\mathrm{\alpha }}\left( q \right)|{\mathrm{\Phi }}_{\mathrm{G}}\rangle + \eta _{{\mathrm{LE}}}^{\mathrm{\alpha }}\left( q \right)|{\mathrm{\Phi }}_{{\mathrm{LE}}}\rangle + \eta _{{\mathrm{D}}^ + {\mathrm{A}}^ - }^{\mathrm{\alpha }}\left( q \right)|{\mathrm{\Phi }}_{{\mathrm{D}}^ + {\mathrm{A}}^ - }\rangle$$

Here, *η*(*q*) denotes the expansion coefficients. The electronic wavefunctions $|{\mathrm{\Phi }}_{{\mathrm{G}}/{\mathrm{LE}}/{\mathrm{D}}^ + {\mathrm{A}}^ - }\rangle$ are the eigenfunctions of the electronic Hamiltonian with fixed nuclear coordinates at the reference geometry (e.g., here, the equilibrium position (*q* = 0) of the G state is selected as the reference point, see Fig. 1):

7 $$\hat H_{\mathrm{e}}(q = 0)|{\mathrm{\Phi }}_{{\mathrm{G}}/{\mathrm{LE}}/{\mathrm{D}}^ + {\mathrm{A}}^ - }\rangle = E_{{\mathrm{G}}/{\mathrm{LE}}/{\mathrm{D}}^ + {\mathrm{A}}^ - }(q = 0)|{\mathrm{\Phi }}_{{\mathrm{G}}/{\mathrm{LE}}/{\mathrm{D}}^ + {\mathrm{A}}^ - }\rangle$$

The eigenenergies $E_{{\mathrm{G}}/{\mathrm{LE}}/{\mathrm{D}}^ + {\mathrm{A}}^ - }(q)$ are then expanded with respect to small nuclear displacements around the reference point. In the harmonic approximation and using the dimensionless normal coordinates *q*_*i*_, the eigenenergies $E_{{\mathrm{G}}/{\mathrm{LE}}/{\mathrm{D}}^ + {\mathrm{A}}^ - }(q)$ are given as:

8 $$E_{\mathrm{G}}\left( {q_{\mathrm{i}}} \right) = {\sum }_i \frac{{\hbar \omega _{\mathrm{i}}}}{2}q_{\mathrm{i}}^2$$

9 $$E_{{\mathrm{LE}}}\left( {q_{\mathrm{i}}} \right) = E_{{\mathrm{LE}}}\left( {q_{\mathrm{i}} = 0} \right) + {\sum }_i \sqrt 2 g_{\mathrm{i}}^{{\mathrm{LE}}}\hbar \omega _{\mathrm{i}}q_{\mathrm{i}} + {\sum }_i \frac{{\hbar \omega _{\mathrm{i}}}}{2}q_{\mathrm{i}}^2$$

10 $$E_{{\mathrm{D}}^ + {\mathrm{A}}^ - }\left( {q_{\mathrm{i}}} \right) = E_{{\mathrm{D}}^ + {\mathrm{A}}^ - }\left( {q_{\mathrm{i}} = 0} \right) + {\sum }_i \sqrt 2 g_{\mathrm{i}}^{{\mathrm{D}}^ + {\mathrm{A}}^ - }\hbar \omega _{\mathrm{i}}q_{\mathrm{i}} + {\sum }_i \frac{{\hbar \omega _{\mathrm{i}}}}{2}q_{\mathrm{i}}^2$$

Here, $\sqrt 2 g_i\hbar \omega _{\mathrm{i}}$ is the linear electron-vibration coupling associated with the *i*th vibrational normal mode with energy ℏ*ω*_i_, which is defined by $\left. {\partial E(q_{\mathrm{i}})/\partial q_{\mathrm{i}}} \right|_{q_{\mathrm{i}} = 0}$; thus, the relaxation energies *λ* of the D^+^A^−^ and LE states (see Fig. 1) are directly related to the vibronic coupling constants via $\lambda = \mathop {\sum }\limits_{\mathrm{i}} g_{\mathrm{i}}^2\hbar \omega _{\mathrm{i}}$. $E_{{\mathrm{LE}}/{\mathrm{D}}^ + {\mathrm{A}}^ - }\left( {q_{\mathrm{i}} = 0} \right)$ is the vertical excitation energy from the G state to the LE/D^+^A^−^ state, which is defined as the sum between the relaxed energy $E_{{\mathrm{LE}}/{\mathrm{D}}^ + {\mathrm{A}}^ - }^0$ of the LE/D^+^A^−^ state and the relaxation energy $\lambda _{{\mathrm{LE}}/{\mathrm{D}}^ + {\mathrm{A}}^ - }$ (see Fig. 1). Thus, the vibronic Hamiltonian matrix that accounts for linear electron-vibration couplings reads:^42,54,60^

11 $${{{\mathbf{H}}_{{\mathrm{vib}}}} = {\left[ {\begin{array}{*{20}{c}} {{\sum }_i \frac{{\hbar \omega _{\mathrm{i}}}}{2}\left( {p_{\mathrm{i}}^2 + q_{\mathrm{i}}^2} \right)} & 0 & 0 \\ 0 & {{\sum }_i \frac{{\hbar \omega _{\mathrm{i}}}}{2}\left( {p_{\mathrm{i}}^2 + q_{\mathrm{i}}^2} \right)} & 0 \\ 0 & 0 & {{\sum }_i \frac{{\hbar \omega _{\mathrm{i}}}}{2}\left( {p_{\mathrm{i}}^2 + q_{\mathrm{i}}^2} \right)} \end{array}} \right]}} \\ \hskip 27pt{{ + \left[ {\begin{array}{*{20}{c}} 0 & 0 & {t_{{\mathrm{D}}^ + {\mathrm{A}}^ - - {\mathrm{G}}}} \\ 0 & {E_{{\mathrm{LE}}}^0 + \lambda _{{\mathrm{LE}}} + {\sum }_i \sqrt 2 g_{\mathrm{i}}^{{\mathrm{LE}}}\hbar \omega _{\mathrm{i}}q_{\mathrm{i}}} & {t_{{\mathrm{D}}^ + {\mathrm{A}}^ - - {\mathrm{LE}}}} \\ {t_{{\mathrm{D}}^ + {\mathrm{A}}^ - - {\mathrm{G}}}} & {t_{{\mathrm{D}}^ + {\mathrm{A}}^ - - {\mathrm{LE}}}} & {E_{{\mathrm{D}}^ + {\mathrm{A}}^ - }^0 + \lambda _{{\mathrm{D}}^ + {\mathrm{A}}^ - } + {\sum }_i \sqrt 2 g_{\mathrm{i}}^{{\mathrm{D}}^ + {\mathrm{A}}^ - }\hbar \omega _{\mathrm{i}}q_{\mathrm{i}}} \end{array}} \right]}}$$

Here, $t_{{\mathrm{D}}^ + {\mathrm{A}}^ - - {\mathrm{G}}}$ and $t_{{\mathrm{D}}^ + {\mathrm{A}}^ - - {\mathrm{LE}}}$ denote the electronic couplings of the D^+^A^−^ state with the G state and LE state, respectively; *p*_i_ corresponds to the dimensionless momentum of the *i*th vibrational normal mode.

The full dynamic solution of the vibronic Hamiltonian given by Eq. (11) can be obtained only numerically, by expanding the coefficients *η*(*q*) in Eq. (6) in terms of the complete set of harmonic oscillator eigenfunctions, |*χ*_k_(*q*_i_)〉^42,54,60^:

12 $$\begin{array}{*{20}{l}} \Psi _{\mathrm{\alpha }} \hfill & = \hfill & {|{\mathrm{\Phi }}_{\mathrm{G}}\rangle \mathop {\sum }\limits_{{\mathrm{m}},{\mathrm{n}}, \ldots ,{\mathrm{k}}} c_{{\mathrm{G}};{\mathrm{m}},{\mathrm{n}}, \ldots ,{\mathrm{k}}}^{\mathrm{\alpha }}|\chi _{\mathrm{m}}(q_1)\rangle |\chi _{\mathrm{n}}(q_2)\rangle \ldots |\chi _{\mathrm{k}}(q_{\mathrm{i}})\rangle } \hfill \\ {} \hfill & {} \hfill & { + |{\mathrm{\Phi }}_{{\mathrm{LE}}}\rangle \mathop {\sum }\limits_{{\mathrm{m}},{\mathrm{n}}, \ldots ,{\mathrm{k}}} c_{{\mathrm{LE}};{\mathrm{m}},{\mathrm{n}}, \ldots ,{\mathrm{k}}}^{\mathrm{\alpha }}|\chi _{\mathrm{m}}(q_1)\rangle |\chi _{\mathrm{n}}(q_2)\rangle \ldots |\chi _{\mathrm{k}}(q_{\mathrm{i}})\rangle } \hfill \\ {} \hfill & {} \hfill & { + |{\mathrm{\Phi }}_{{\mathrm{D}}^ + {\mathrm{A}}^ - }\rangle \mathop {\sum }\limits_{{\mathrm{m}},{\mathrm{n}}, \ldots ,{\mathrm{k}}} c_{{\mathrm{D}}^ + {\mathrm{A}}^ - ;{\mathrm{m}},{\mathrm{n}}, \ldots ,{\mathrm{k}}}^{\mathrm{\alpha }}|\chi _{\mathrm{m}}(q_1)\rangle |\chi _{\mathrm{n}}(q_2)\rangle \ldots |\chi _{\mathrm{k}}(q_{\mathrm{i}})\rangle } \hfill \end{array}$$

Each adiabatic solution *Ψ*_α_ is the superposition of the |Φ_G_〉, |Φ_LE_〉 and $|{\mathrm{\Phi }}_{{\mathrm{D}}^ + {\mathrm{A}}^ - }\rangle$ diabatic states where the *c*_*i*_ terms are the expansion coefficients. By using a finite but large enough number of vibrational functions (large *k*), the eigenenergies and eigenfunctions of the vibronic Hamiltonian can be obtained with any desirable accuracy.

Based on the calculated eigenenergies and eigenfunctions of the vibronic Hamiltonian, the optical absorption intensity (directly proportional to the extinction coefficient) per donor/acceptor pair is obtained from:

13 $$A(E) = E\mathop {\sum }\limits_{\mathrm{\beta }} \mathop {\sum }\limits_{\mathrm{\alpha }} \left[ {f\left( {E_{\mathrm{\alpha }}} \right) - f\left( {E_{\mathrm{\beta }}} \right)} \right]\left| {\left\langle {\Psi _{\mathrm{\alpha }}{\mathrm{|}}\overrightarrow {\mathbf{e}} \cdot \overrightarrow {\mathbf{\mu}} {\mathrm{|}}\Psi _{\mathrm{\beta }}} \right\rangle } \right|^2\delta (E - (E_{\mathrm{\beta }} - E_{\mathrm{\alpha }}))$$

where *f*(*E*_α_) stands for the thermal (Boltzmann) population of the vibronic state *E*_α_; $\overrightarrow {\mathbf{e}}$, the polarization of the electric field; and $\overrightarrow {\mathbf{\mu}}$, is the dipole moment operator. In actual calculations, the delta function is replaced with a Gaussian function whose width is taken to be the same for all vibronic contributions^54,60^. In the case of vibronic models including a few effective modes, the calculated vibronic peaks are separated by an energy related to that of the lower-energy vibrational mode. In these instances, it is a standard practice to use a Gaussian smearing of the vibronic peaks. In order to obtain a smooth profile of the simulated absorption spectra, we used a Gaussian broadening with a width (100 cm^−1^) set according to the energy of the low-frequency vibration (100 cm^−1^). We note that this procedure has a negligible impact on the overall width and asymmetry of the simulated absorption spectra. The transition dipole moment, *μ*_CT_, of the CT absorption can be then obtained via the integration of *A*(*E*)/*E*^54^.

Without any loss of generality, we assume that the LE state is localized on the donor and that the transition dipole moment $\left( {\overrightarrow {\mathbf{\mu }} _{\mathrm{l}}} \right)$ of this state is oriented along the long axis of the molecule as shown in Supplementary Fig. 6. The choice of the system of coordinates, the orientation of the donor molecule, and the orientation of the electrical polarization of the incident light are also illustrated in Supplementary Fig. 6; the direction of interfacial electron transfer from donor to acceptor is chosen to be parallel to the *X* axis, and the difference between the dipole moments of the G and D^+^A^−^ states is given by $e{\mathop{{\mathbf{R}}}\limits^{\rightarrow}}_{{\mathrm{ET}}}$^35^, where ${\mathop{{\mathbf{R}}}\limits^{\rightarrow}}_{{\mathrm{ET}}}$ is the diabatic electron transfer distance.

## Supplementary Information

Supplementary Information

## Data Availability

The numerical code and the data that support the findings of this study are available from the corresponding authors upon request.
