# Supplementary material for: Assessing the nature of the charge-transfer electronic states in organic solar cells
Source: Nat Commun. 2018 Dec 13;9:5295. doi: 10.1038/s41467-018-07707-8 (PMC6294259; doi:10.1038/s41467-018-07707-8)
Supplement: Supplementary file 1 — Supplementary Information [file 41467_2018_7707_MOESM1_ESM.docx]

**Supplementary Information**

**Assessing the Nature of the Charge-Transfer Electronic States in Organic Solar Cells**

Chen *et al*.

**Supplementary Figures**

**
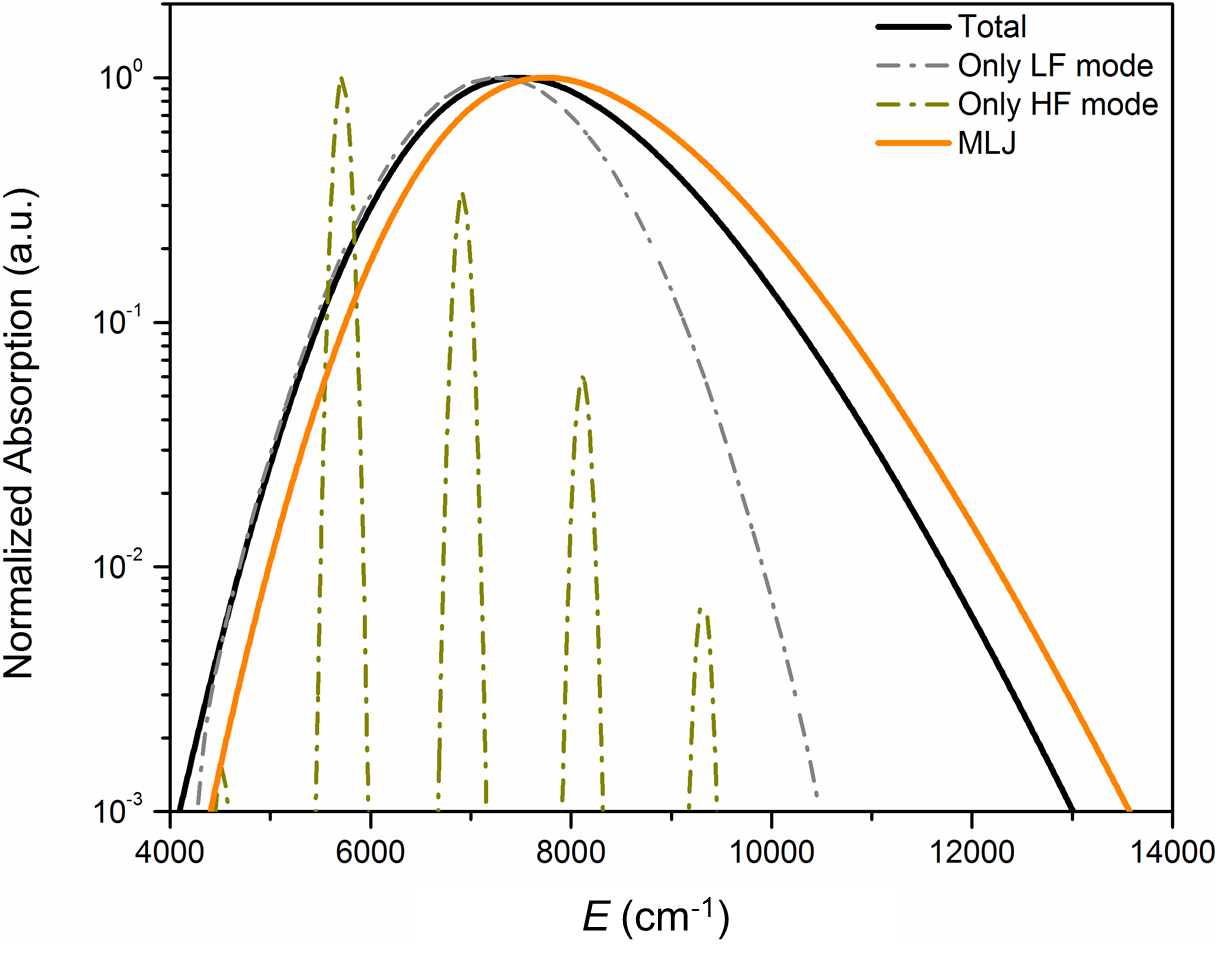
**

**Supplementary Figure 1**. CT absorption bands simulated by our vibronic model and by the Marcus-Levich-Jortner (MLJ) formula; here, the same electronic-structure parameters are used in the two approaches. ‘Total’ denotes the spectra simulated by our vibronic model that considers both low- and high-frequency (LF and HF) vibrational modes; ‘Only LF [HF] mode’ denotes the spectra simulated by the vibronic model when considering only the low- [high-] frequency vibrational mode.

**
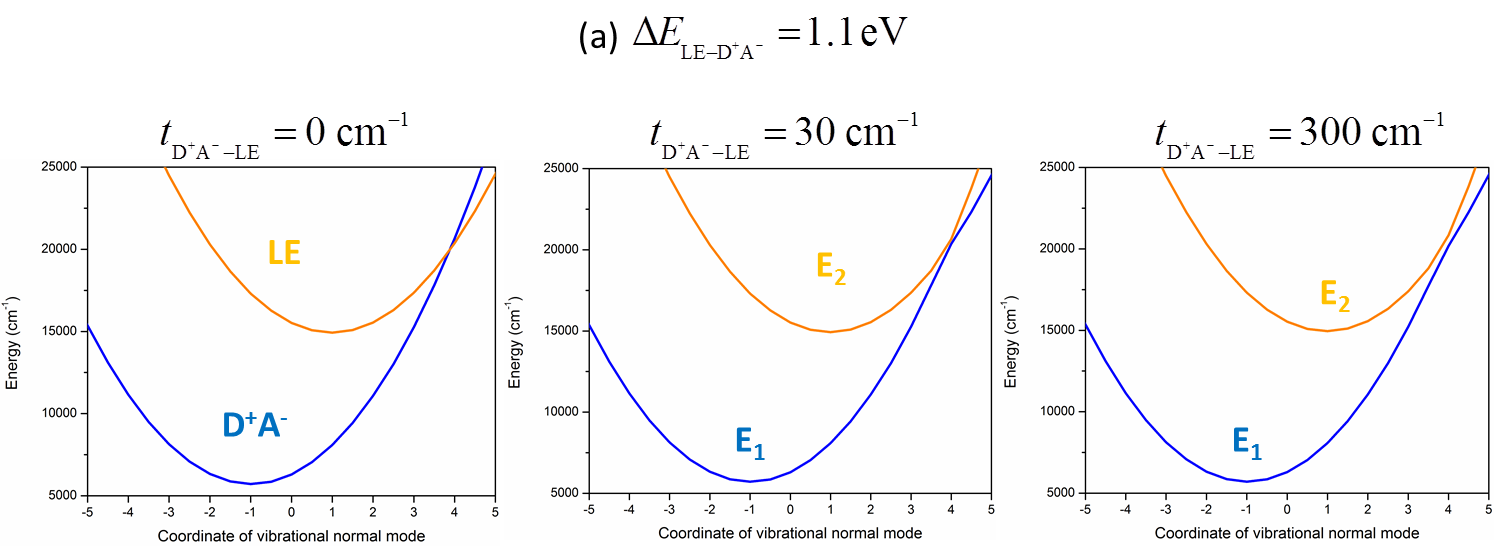
**

**
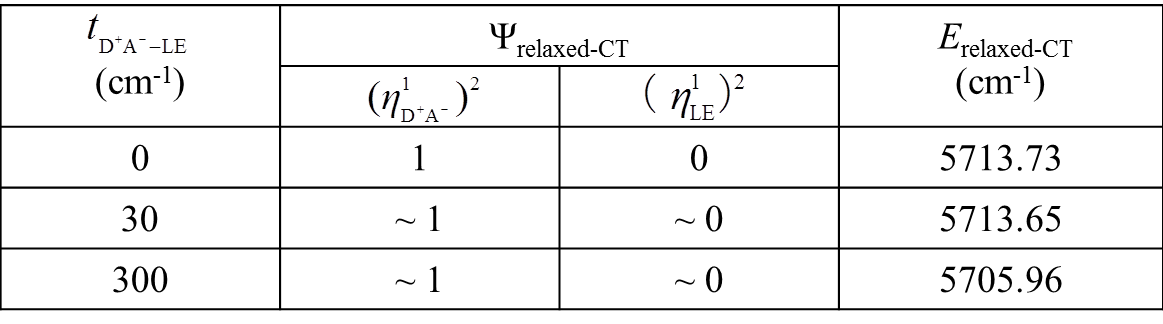
**

**
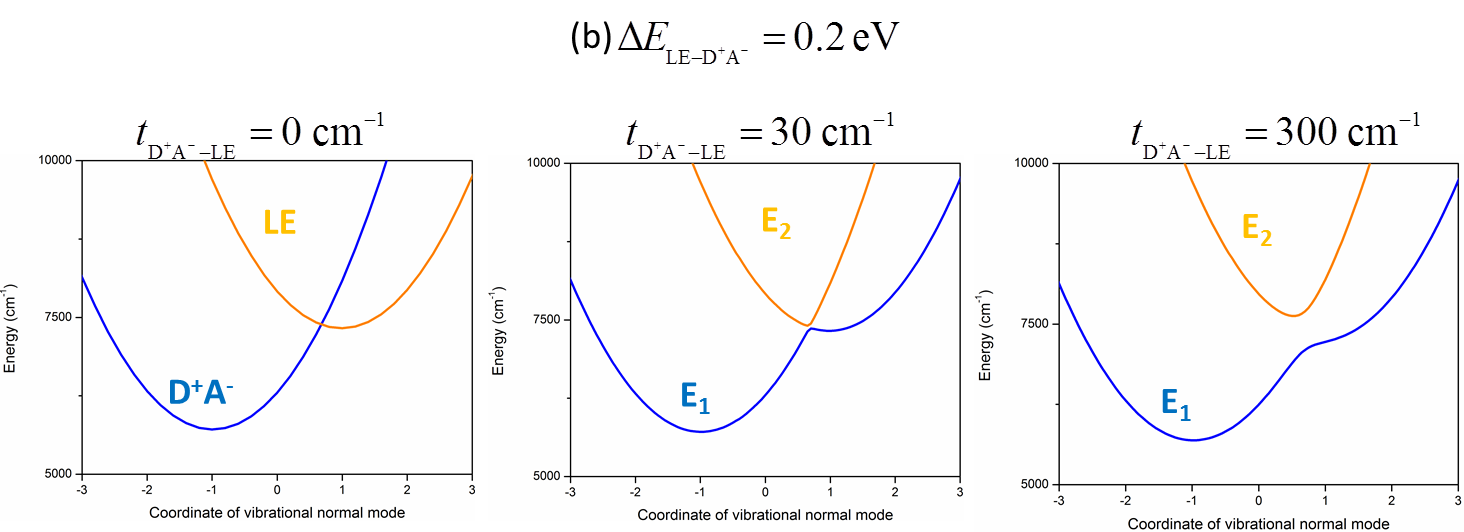
**

**
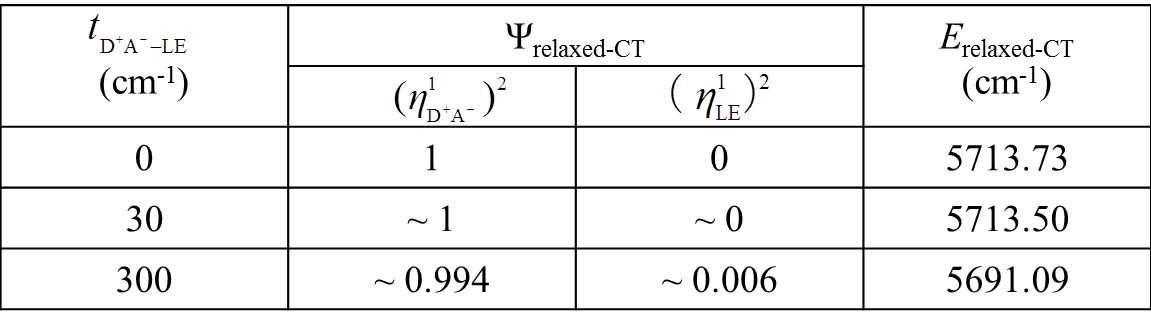
**

**
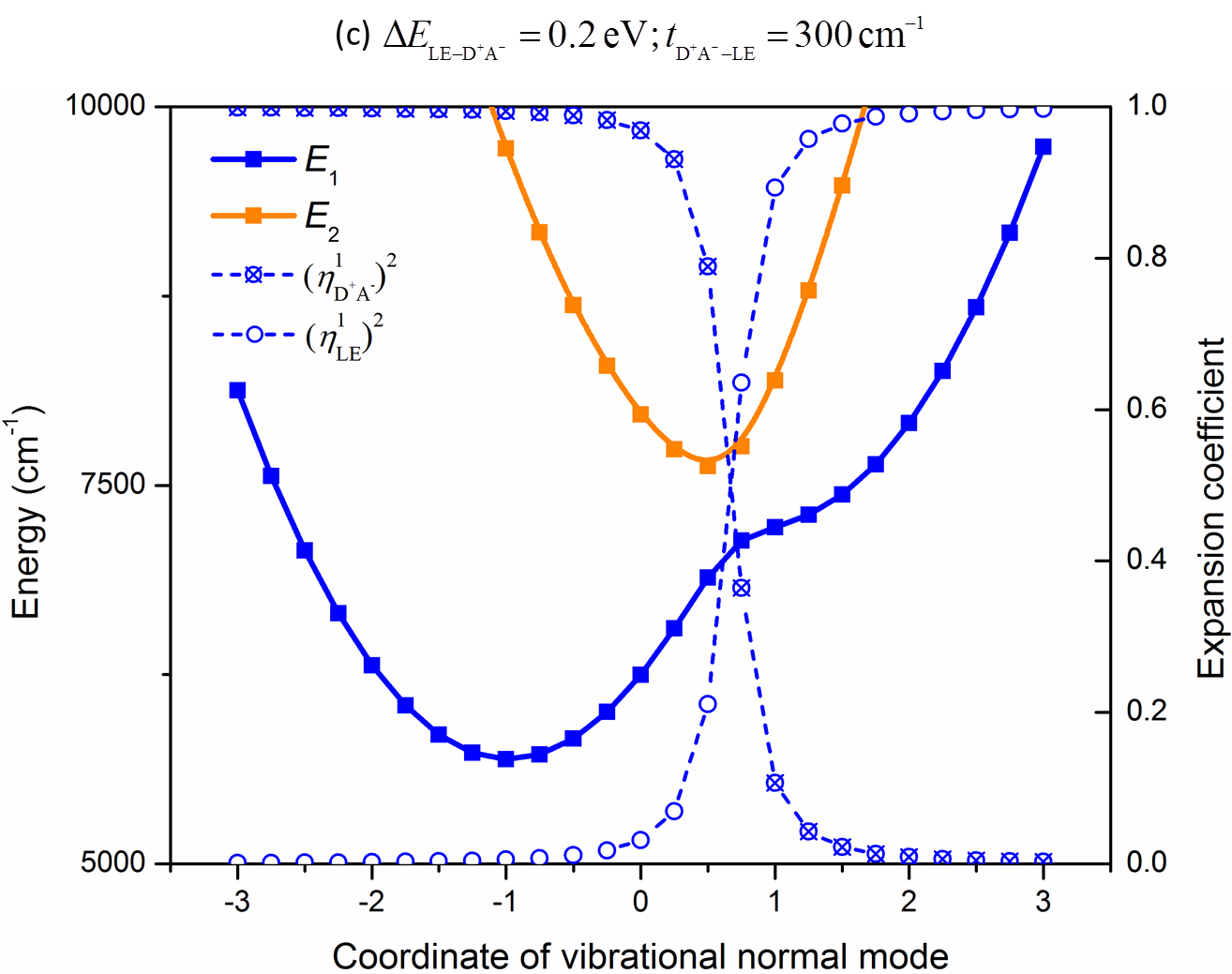
**

**Supplementary Figure 2. (a)** Upper panel: Diagram of the potential energy surfaces for the adiabatic states (E_1_ and E_2_) in the case of $\text{Δ}E_{\text{LE}-\text{D}^{+}\text{A}^{-}}$ = 1.1 eV (8871 cm^-1^), when $t_{\text{D}^{+}\text{A}^{-}-\text{LE}}$ is equal to 0, 30, and 300 cm^-1^, respectively; lower panel: Energies (*E*_relaxed-CT_) and expansion coefficients ($(\eta_{\text{LE}}^{1})^{2}$ and $\text{(}\eta_{\text{D}^{+}\text{A}^{-}}^{1}\text{)}^{2}$) of the wavefunctions (Ψ_relaxed-CT_) corresponding to the relaxed CT states. **(b)** Upper panel: Diagram of the potential energy surfaces for the adiabatic states (E_1_ and E_2_) in the case of $\text{Δ}E_{\text{LE}-\text{D}^{+}\text{A}^{-}}$ = 0.2 eV (1613 cm^-1^), when $t_{\text{D}^{+}\text{A}^{-}-\text{LE}}$ is equal to 0, 30, and 300 cm^-1^, respectively; lower panel: Energies (*E*_relaxed-CT_) and expansion coefficients ($(\eta_{\text{LE}}^{1})^{2}$ and $\text{(}\eta_{\text{D}^{+}\text{A}^{-}}^{1}\text{)}^{2}$) of the wavefunctions (Ψ_relaxed-CT_) corresponding to the relaxed CT states. **(c)** Expansion coefficients ($(\eta_{\text{LE}}^{1})^{2}$ and $\text{(}\eta_{\text{D}^{+}\text{A}^{-}}^{1}\text{)}^{2}$) of the wavefunctions corresponding to the lower state (E_1_) as a function of the coordinates of the vibrational normal mode.

**
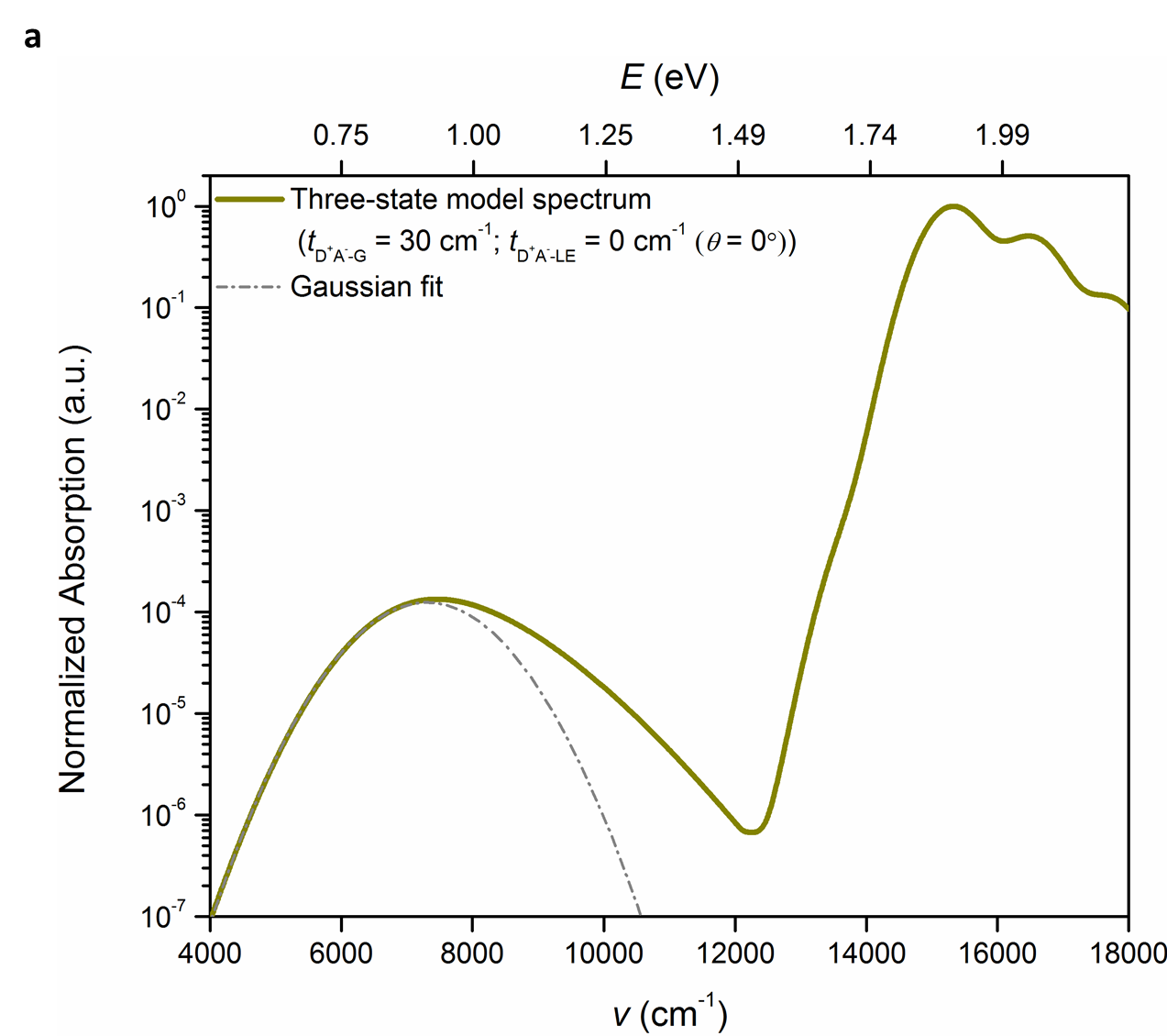
**

**
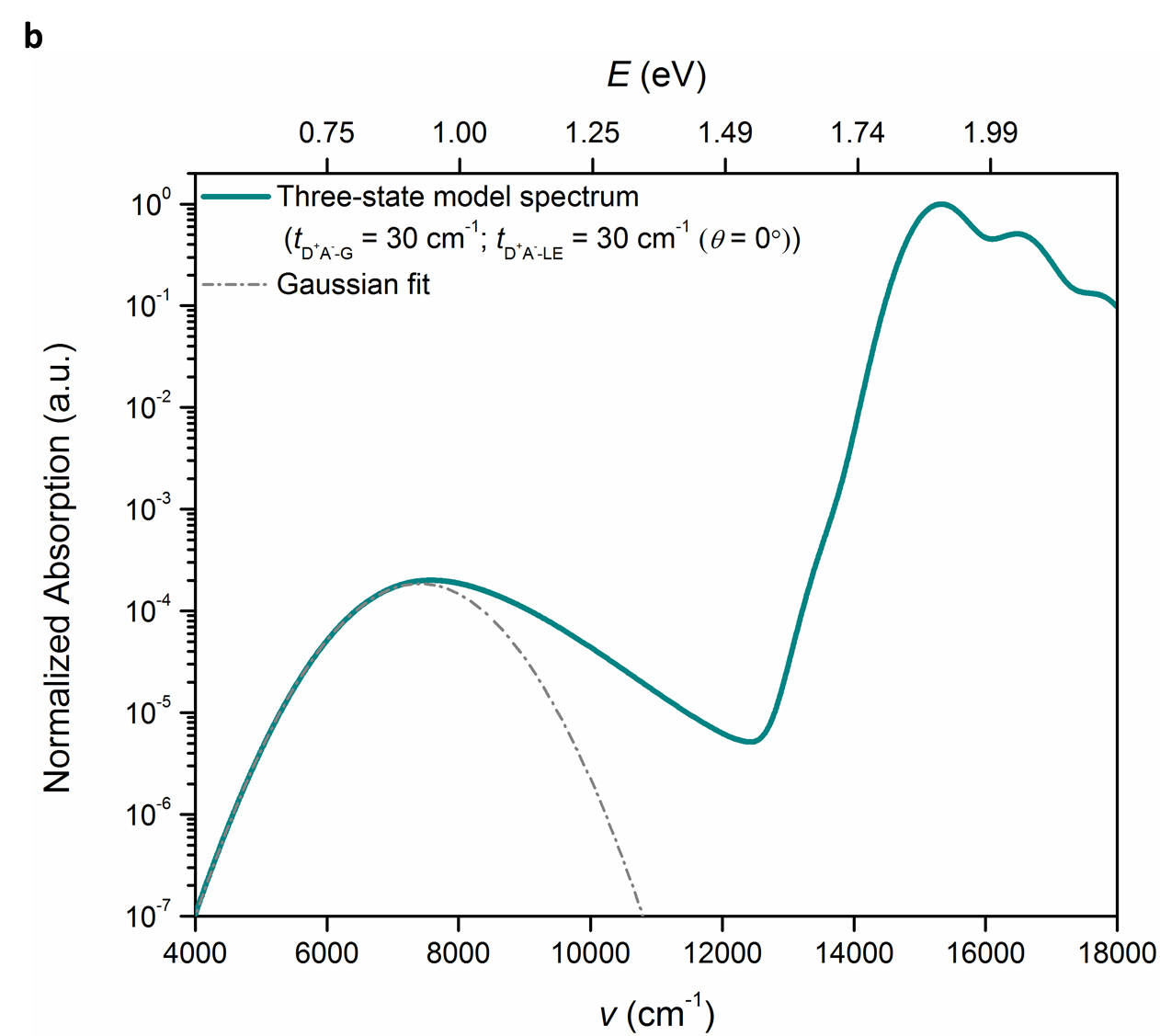
**

**Supplementary Figure 3.** (a) and (b) Gaussian-fit to the low-energy absorption tails of the CT absorption bands simulated via the three-state vibronic approach for the configuration in the case where $\Delta E_{\text{LE}-\text{D}^{+}\text{A}^{-}}$= 1.1 eV (8871 cm^-1^); the photon polarization is taken parallel to the X axis (*γ* = 0°), see Supplementary Figure 6. *E*, photon energy; *v*, wavenumber.

**
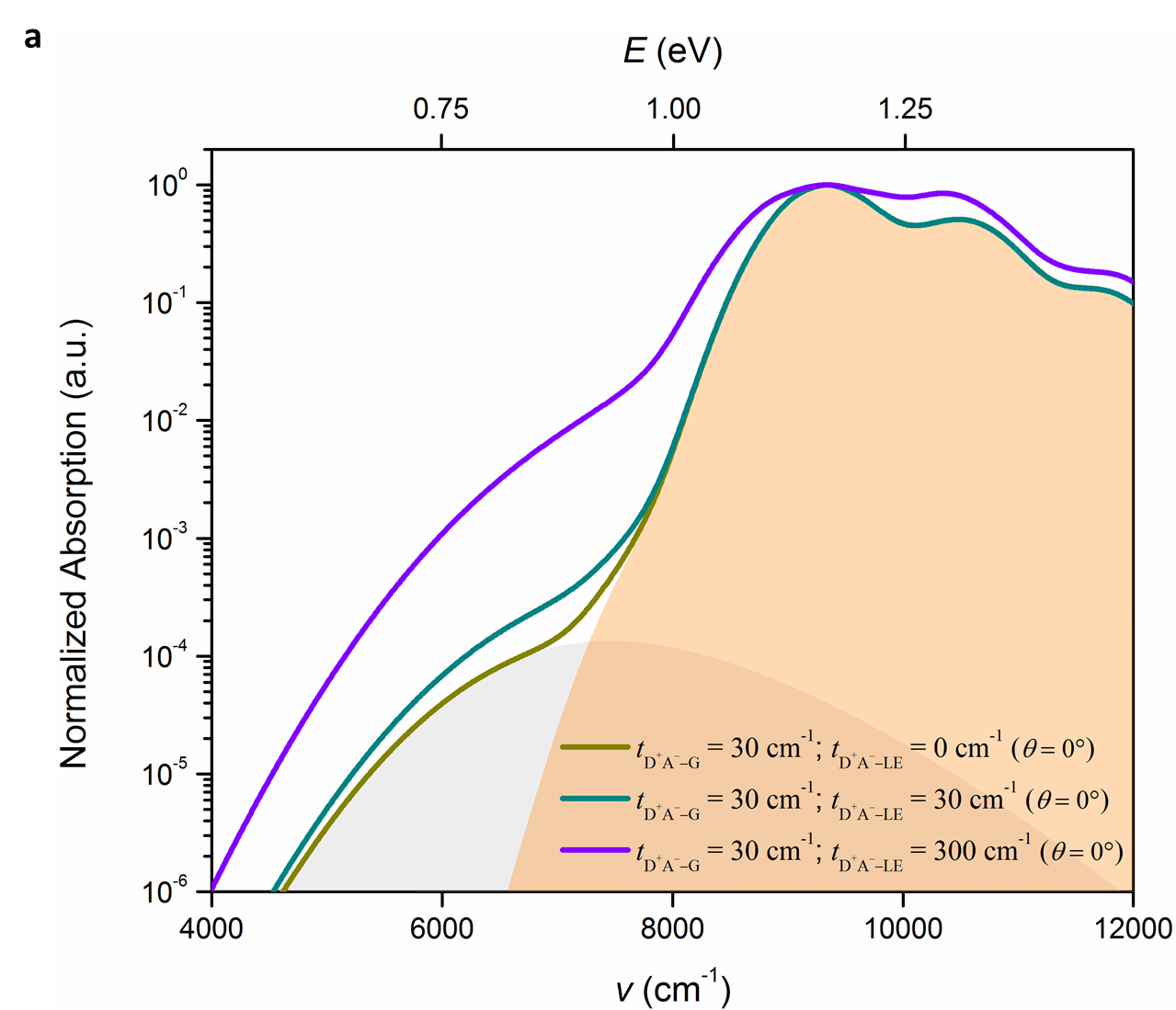
**

**
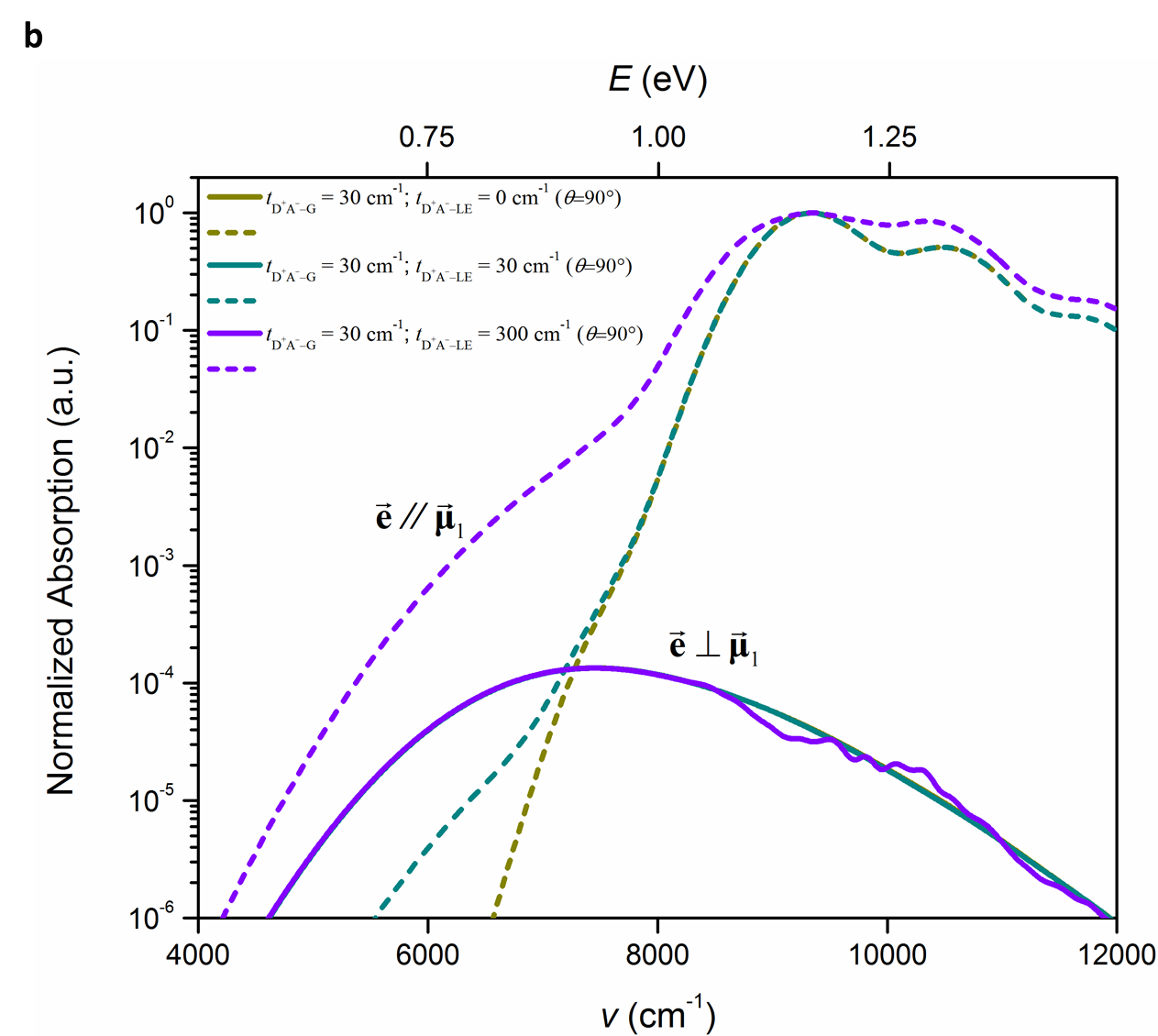
**

**Supplementary Figure 4.** (**a**) Absorption spectra simulated via the three-state vibronic model for the configuration in the case of the $\Delta E_{\text{LE}-\text{D}^{+}\text{A}^{-}}$= 0.4 eV (3226 cm^-1^). The regions filled in gray and orange correspond to the D^+^A^-^ and LE absorption bands, respectively, in the absence of any D^+^A^-^ -LE coupling ($t_{\text{D}^{+}\text{A}^{-}-\text{LE}}=0$ cm^-1^). (**b**) Absorption spectra simulated via the three-state vibronic model for the configuration in the case of the $\Delta E_{\text{LE}-\text{D}^{+}\text{A}^{-}}$= 0.4 eV (3226 cm^-1^). The solid and dashed lines correspond to the simulated absorption spectra in the cases where and (see Supplementary Figure 6), respectively. *E*, photon energy; *v*, wavenumber.

**
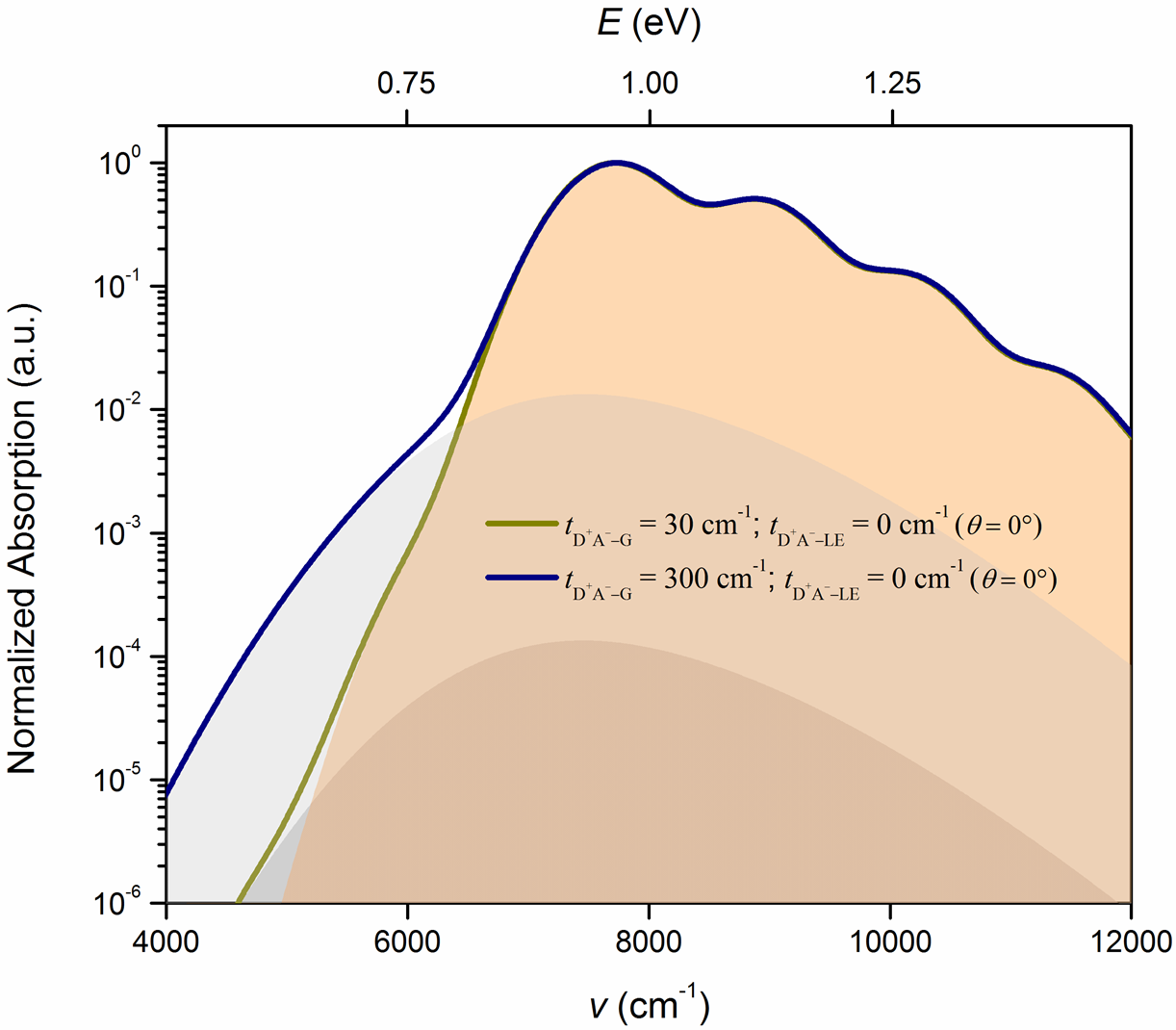
**

**Supplementary Figure 5.** Absorption spectra simulated via the three-state vibronic model for the configuration, in the case of $\text{Δ}E_{\text{LE}-\text{D}^{+}\text{A}^{-}}$= 0.2 eV (1613 cm^-1^), when the photon polarization is parallel to the X axis (*γ* = 0°). The regions filled in gray and orange correspond to the D^+^A^-^ and LE absorption bands, respectively, in the absence of any D^+^A^-^ -LE coupling ($t_{\text{D}^{+}\text{A}^{-}-\text{LE}}=0$ cm^-1^). *E*, photon energy; *v*, wavenumber.

**
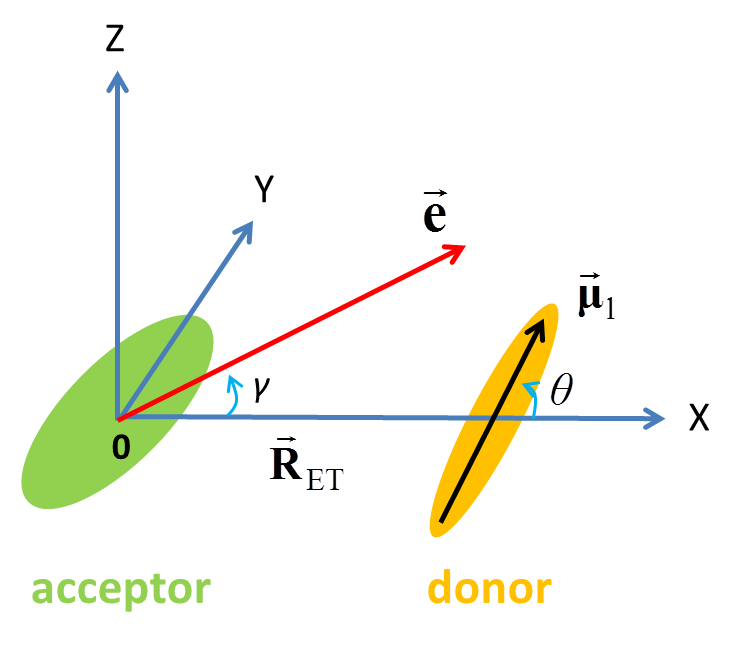
**

**
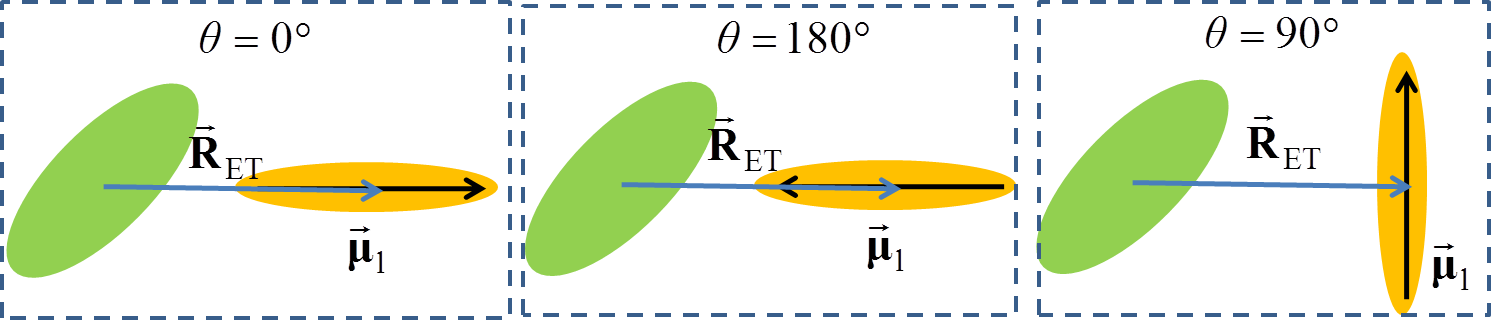
**

**Supplementary Figure 6.** Schematic diagram of the molecular orientations and direction of photon polarization. *θ* denotes the angle between the donor molecular long axis and the X direction; *γ*, the angle between photon polarization and the X axis; $\vec{\mathbf{e}}$, the polarization of the electric field; $\vec{\mathbf{R}}_{\text{ET}}$, the diabatic electron-transfer (ET) distance between donor and acceptor; and $\vec{\mathbf{\mu}}_{\text{l}}$, the transition dipole of the LE state localized on the donor.

**Supplementary Tables**

**Supplementary Table 1**. Parameters used in the vibronic simulation of the absorption spectrum of the PBTTT/PCBM blend.

| Parameter | Value |
| --- | --- |
| Relaxed excitation energy of the LE state, $E_{\text{LE}}^{0}$ | 2.00 eV (16130 cm^-1^) |
| Relaxed excitation energy of the D^+^A^-^ state, $E_{\text{D}^{+}\text{A}^{-}}^{0}$ | 1.04 eV  (8387 cm^-1^) |
| Energy of the high-frequency (HF)  vibrational normal mode, | 1400 cm^-1^ |
| Vibronic coupling constant corresponding to  the LE state for the HF vibrational mode,$g_{\text{HF}}^{\text{LE}}$ | 1.02 |
| Vibronic coupling constant corresponding to  the D^+^A^-^ state for the HF vibrational mode, $g_{\text{HF}}^{\text{D}^{+}\text{A}^{-}}$ | 1.02 |
| Energy of the low-frequency (LF)  vibrational normal mode, | 100 cm^-1^ |
| Vibronic coupling constant corresponding to  the LE state for the LF vibrational mode,$g_{\text{LF}}^{\text{LE}}$ | 1.9 |
| Vibronic coupling constant corresponding to  the D^+^A^-^ state for the LF vibrational mode,$g_{\text{LF}}^{\text{D}^{+}\text{A}^{-}}$ | 6.0 |
| Transition dipole of the LE state localized on the donor, | 10 D |
| Diabatic electron-transfer (ET) distance between donor and acceptor, | 7.2 Å |
| D^+^A^-^-G electronic coupling, $t_{\text{D}^{+}\text{A}^{-}-\text{G}}$ | 300 cm^-1^ |
| D^+^A^-^-LE electronic coupling, $t_{\text{D}^{+}\text{A}^{-}-\text{LE}}$ | 450 cm^-1^ |

**Supplementary Table 2**. Parameters used in the vibronic simulations of the absorption spectra of the PIPCP neat film and the PIPCP/PCBM blend.

| Parameter | Value |
| --- | --- |
| Relaxed excitation energy of the LE state, $E_{\text{LE}}^{0}$ | 1.49 eV  (12016 cm^-1^) |
| Relaxed excitation energy of the D^+^A^-^ state, $E_{\text{D}^{+}\text{A}^{-}}^{0}$ | 1.29 eV  (10403 cm^-1^) |
| Energy of the high-frequency (HF)  vibrational normal mode, | 1400 cm^-1^ |
| Vibronic coupling constant corresponding to  the LE state for the HF vibrational mode,$g_{\text{HF}}^{\text{LE}}$ | 0.85 |
| Vibronic coupling constant corresponding to  the D^+^A^-^ state for the HF vibrational mode, $g_{\text{HF}}^{\text{D}^{+}\text{A}^{-}}$ | 0.85 |
| Energy of the low-frequency (LF)  vibrational normal mode, | 100 cm^-1^ |
| Vibronic coupling constant corresponding to  the LE state for the LF vibrational mode,$g_{\text{LF}}^{\text{LE}}$ | 2.8 |
| Vibronic coupling constant corresponding to  the D^+^A^-^ state for the LF vibrational mode,$g_{\text{LF}}^{\text{D}^{+}\text{A}^{-}}$ | 4.0 |
| Transition dipole of the LE state localized on the donor, | 10 D |
| Diabatic electron-transfer (ET) distance between donor and acceptor, | 8.3 Å |
| D^+^A^-^-G electronic coupling, $t_{\text{D}^{+}\text{A}^{-}-\text{G}}$ | 100 cm^-1^ |
| D^+^A^-^-LE electronic coupling, $t_{\text{D}^{+}\text{A}^{-}-\text{LE}}$ | 400 cm^-1^ |

**Supplementary Table 3**. DFT-calculated electronic couplings evaluated at the ωB97XD/6-31G(d,p) level with non-empirically tuned ω values for the PIPCP/PCBM molecular complexes. Here, ‘H’, ‘L’, and ‘L+1’ denote the HOMO, LUMO and LUMO+1 orbitals, respectively; ‘D’ and ‘A’ denote the PIPCP donor and PCBM acceptor, respectively.

| Configuration | D^+^A^-^-LE | | D^+^A^-^-G | |
| --- | --- | --- | --- | --- |
|  | *t*_L(D)-L(A)_  (cm^-1^) | *t*_L(D)-L+1(A)_  (cm^-1^) | *t*_H(D)-L(A)_  (cm^-1^) | *t*_H(D)-L+1(A)_  (cm^-1^) |
| 1 | 429.6 | 231.8 | 108.2 | 266.2 |
| 2 | 81.3 | 53.8 | 23.6 | 254.5 |
| 3 | 19.5 | 52.0 | 92.7 | 226.9 |
| 4 | 239.5 | 129.2 | 21.7 | 79.3 |
| 5 | 118.7 | 383.1 | 64.4 | 35.6 |
| 6 | 333.5 | 186.0 | 73.5 | 65.6 |
| 7 | 106.1 | 151.4 | 304.0 | 142.5 |
| 8 | 102.2 | 186.0 | 64.8 | 17.9 |

**Supplementary Discussions**

**Supplementary Discussion 1:** Detailed discussion on the parameters chosen in the three-state model study

Here, we discuss in more detail the choice of the parameters used in the three-state model. In order to prevent prohibitive computational costs while capturing the physical nature of the problem, a common approximation in the theory of vibronic coupling is to use effective vibrational normal modes;[^1^](#_ENREF_1) here, our vibronic model takes into account two effective vibrational modes: a high-frequency (HF) vibration mode ( = 1200 cm^-1^), typical of a carbon-carbon bond stretch[^2^](#_ENREF_2), [^3^](#_ENREF_3), and a low-frequency (LF) vibration mode ( = 100 cm^-1^), which represents rotations between intramolecular fragments and intermolecular motions[^4^](#_ENREF_4), [^5^](#_ENREF_5). For the LE state on the donor, the vibronic coupling constant $g_{\text{HF}}^{\text{LE}}$ related with the HF mode is set as 0.7 and the $g_{\text{LF}}^{\text{LE}}$ related with the LF mode is taken as 2;[^4^](#_ENREF_4) these values are chosen in such a way that the relaxation energy of the LE state $\lambda_{\text{LE}}$is *ca*. 0.12 eV (988 cm^-1^), which is representative of typical organic semiconductor materials.[^4^](#_ENREF_4), [^6^](#_ENREF_6) For the D^+^A^-^ state, the $g_{\text{HF}}^{\text{D}^{+}\text{A}^{-}}$ and $g_{\text{LF}}^{\text{D}^{+}\text{A}^{-}}$ values are set at 0.7 and 4.2, respectively; this results in a relaxation energy $\lambda_{\text{D}^{+}\text{A}^{-}}$= *ca*. 0.3 eV (2352 cm^-1^), which is consistent with earlier calculations.[^7-10^](#_ENREF_7) Here, we recall that the vibronic coupling constant $g_{\text{HF}}$ related to the intrinsic high-frequency C-C bond stretching in either the D^+^A^-^ or LE state is usually on the order of < 1. On the other hand, the $g_{\text{LF}}^{\text{D}^{+}\text{A}^{-}}$ value related to the low-frequency intermolecular vibration is larger for the D^+^A^-^ state than $g_{\text{LF}}^{\text{LE}}$ for the LE state. Indeed, intermolecular motions significantly impact the intermolecular polarization interactions and the D⁺A⁻-state dipole moment, and thus substantially modifies the D⁺A⁻-state energy,[^11^](#_ENREF_11) which eventually leads to a large vibronic coupling constant.[^12^](#_ENREF_12) In the present work, the average D^+^A^-^-G electronic coupling $t_{\text{D}^{+}\text{A}^{-}-\text{G}}$ is taken as 30 cm^-1^, which is consistent with the fact that most of the efficient OSC material systems have small $t_{\text{D}^{+}\text{A}^{-}-\text{G}}$ values, usually smaller than 100 cm^-1^;[^9^](#_ENREF_9), [^13^](#_ENREF_13), [^14^](#_ENREF_14) the D^+^A^-^-LE electronic coupling $t_{\text{D}^{+}\text{A}^{-}-\text{LE}}$ is varied within the range from 0 to 300 cm^-1^,[^15^](#_ENREF_15), [^16^](#_ENREF_16) in order to evaluate its impact on the absorption spectra.

**Supplementary Discussion 2:** Quantum nature of the high-frequency vibration

To illustrate the quantum nature of the high-frequency vibration, we simulate the CT absorption spectrum by our vibronic model that includes either only the high-frequency (HF) or only the low-frequency (LF) vibration, as shown in Supplementary Figure 1. It is found that the absorption spectrum simulated by our vibronic model with inclusion of only the LF vibration is very close to the symmetrical Gaussian lineshape, while the one simulated by our vibronic model with inclusion of only the HF vibration show very clear vibronic peaks, which comes from the quantum nature of the high-frequency vibrations. As a consequence, the ‘total’ absorption spectrum simulated by our vibronic model that include both the HF and LF vibrations is asymmetrical, as shown in Supplementary Figure 1. We also use the Marcus-Levich-Jortner (MLJ) formula[^12^](#_ENREF_12) that treats the high-frequency vibration quantum mechanically to simulate the CT absorption spectrum (see Supplementary Figure 1, where we have taken the same electronic-structure parameters as in our vibronic model). The results indicate that the ‘total’ absorption spectrum simulated by our vibronic model is consistent with that simulated by the MLJ formula.

**Supplementary Discussion 3:** Simple model considering only the D^+^A^-^ and LE states and one vibrational normal mode

In order to provide a clear understanding of the role of the D^+^A^-^-LE coupling in the hybridization of these states, we consider a simple two-state semiclassical vibronic model that accounts only for the D^+^A^-^ and LE states and one vibrational normal mode:

(1)

where the definitions of all the physical quantities can be found in the main text. By solving the eigenequation:

$\mathbf{H}_{\text{vib}}\Psi_{\text{α}}=E_{\text{α}}\Psi_{\text{α}}$ (2)

we obtain its eigenenergy *E*_α_ (α =1, 2) and eigenfunction $\Psi_{\text{α}}$:

$\Psi_{\text{α}}=\eta_{\text{LE}}^{\text{α}}\text{(}q\text{)}|\text{Φ}_{\text{LE}}\rangle+\eta_{\text{D}^{+}\text{A}^{-}}^{\alpha}\text{(}q\text{)}|\text{Φ}_{\text{D}^{+}\text{A}^{-}}\rangle$ (3)

where $\eta\text{(}q\text{)}$ denote the expansion coefficients.

We consider below the solutions of the Hamiltonian (Supplementary Equation 1) for two cases: (a) $\text{Δ}E_{\text{LE}-\text{D}^{+}\text{A}^{-}}$ = 1.1 eV (8871 cm^-1^) and (b) $\text{Δ}E_{\text{LE}-\text{D}^{+}\text{A}^{-}}$ =0.2 eV (1613 cm^-1^). Supplementary Figure 2 shows the adiabatic potential energies when $t_{\text{D}^{+}\text{A}^{-}-\text{LE}}$ is equal to 0, 30, and 300 cm^-1^, respectively. The energies (*E*_relaxed-CT_) and the expansion coefficients ($（\eta_{\text{LE}}^{1}）^{2}$ and $\text{(}\eta_{\text{D}^{+}\text{A}^{-}}^{1}\text{)}^{2}$) of the wavefunctions (Ψ_relaxed-CT_) corresponding to the relaxed CT states are also given in the lower panels of Supplementary Figure 2 (a) and (b). The results show that in all cases, the relaxed CT states are nearly pure CT states (*i.e*., D^+^A^-^ states with a full electron transfer from donor to acceptor). However, as the lower potential energy curve approaches the upper one, the D^+^A^-^-LE coupling leads to a strong hybridization of the D^+^A^-^ and LE states. Supplementary Figure 2 (c) shows the expansion coefficients ($（\eta_{\text{LE}}^{1}）^{2}$ and $\text{(}\eta_{\text{D}^{+}\text{A}^{-}}^{1}\text{)}^{2}$) of the wavefunctions corresponding to the lower state (E_1_) for different coordinates of the vibrational normal mode. For example, for *q* = 0.5 in the case of $\text{Δ}E_{\text{LE}-\text{D}^{+}\text{A}^{-}}$ = 0.2 eV (1613 cm^-1^) and $t_{\text{D}^{+}\text{A}^{-}-\text{LE}}$ = 300 cm^-1^, the wavefunction of the lower state (E_1_) is described by the following composition: $\text{(}\eta_{\text{D}^{+}\text{A}^{-}}^{1}\text{)}^{2}=0.79$ and $\text{（}\eta_{\text{LE}}^{1}\text{）}^{2}=0.21$ (Supplementary Figure 2 (c)). This energy region of the hybrid D^+^A^-^-LE states corresponds to the ‘hot’ vibronic CT states mentioned in the main text and in Fig. 3.

**Supplementary Discussion 4:** DFT-evaluation of the electronic couplings between the D^+^A^-^ and LE/G states in the PIPCP/PCBM molecular pairs

Here, the electronic couplings between the D^+^A^-^ and LE states are approximated by the couplings between the LUMOs of the PIPCP donor and the PCBM acceptor. In PCBM, the LUMO+1 is energetically very close to the LUMO, therefore, the electronic coupling between the PIPCP LUMO and the PCBM LUMO+1 is also evaluated. The electronic couplings between the D^+^A^-^ and G states are approximated by the couplings between the HOMO of the PIPCP and the LUMO or LUMO+1 of the acceptor. The calculations are performed on the basis of the PIPCP/PCBM geometries (1-8) generated in our earlier work.[^17^](#_ENREF_17) The electronic couplings are obtained using the fragment orbital approach,[^5^](#_ENREF_5) and the calculation results are listed in Supplementary Table 3.

**Supplementary References**

1. Köuppel, H., Domcke, W. & Cederbaum, L. S. Multimode Molecular Dynamics Beyond the Born-Oppenheimer Approximation. In: *Advances in Chemical Physics*. John Wiley & Sons, Inc. (2007).

2. Brédas, J.-L., Beljonne, D., Coropceanu, V. & Cornil, J. Charge-Transfer and Energy-Transfer Processes in π-Conjugated Oligomers and Polymers:  A Molecular Picture. *Chem. Rev.* **104**, 4971-5004 (2004).

3. Vandewal, K.*, et al.* Absorption Tails of Donor:C60 Blends Provide Insight into Thermally Activated Charge-Transfer Processes and Polaron Relaxation. *J. Am. Chem. Soc.* **139**, 1699-1704 (2017).

4. Karabunarliev, S. & Bittner, E. R. Polaron–excitons and electron–vibrational band shapes in conjugated polymers. *J. Chem. Phys.* **118**, 4291-4296 (2003).

5. Coropceanu, V., Cornil, J., da Silva Filho, D. A., Olivier, Y., Silbey, R. & Brédas, J.-L. Charge Transport in Organic Semiconductors. *Chem. Rev.* **107**, 926-952 (2007).

6. Sun, H., Zhong, C. & Brédas, J.-L. Reliable Prediction with Tuned Range-Separated Functionals of the Singlet–Triplet Gap in Organic Emitters for Thermally Activated Delayed Fluorescence. *J. Chem. Theory Comput.* **11**, 3851-3858 (2015).

7. Liu, T. & Troisi, A. Absolute Rate of Charge Separation and Recombination in a Molecular Model of the P3HT/PCBM Interface. *J. Phys. Chem. C* **115**, 2406-2415 (2011).

8. Liu, T. & Troisi, A. What Makes Fullerene Acceptors Special as Electron Acceptors in Organic Solar Cells and How to Replace Them. *Adv. Mater.* **25**, 1038-1041 (2013).

9. Chen, X.-K., Wang, T. & Brédas, J.-L. Suppressing Energy Loss due to Triplet Exciton Formation in Organic Solar Cells: The Role of Chemical Structures and Molecular Packing. *Adv. Energy Mater.* **7**, 1602713-1602721 (2017).

10. Chen, X.-K. & Brédas, J.-L. Voltage Losses in Organic Solar Cells: Understanding the Contributions of Intramolecular Vibrations to Nonradiative Recombinations. *Adv. Energy Mater.* **8**, 1702227 (2018).

11. Yang, B., Yi, Y., Zhang, C.-R., Aziz, S. G., Coropceanu, V. & Brédas, J.-L. Impact of Electron Delocalization on the Nature of the Charge-Transfer States in Model Pentacene/C60 Interfaces: A Density Functional Theory Study. *J. Phys. Chem. C* **118**, 27648-27656 (2014).

12. Kahle, F.-J., Rudnick, A., Bässler, H. & Köhler, A. How to interpret absorption and fluorescence spectra of charge transfer states in an organic solar cell. *Mater. Horiz.* **5**, 837-848 (2018).

13. Han, G., Shen, X. & Yi, Y. Deposition Growth and Morphologies of C60 on DTDCTB Surfaces: An Atomistic Insight into the Integrated Impact of Surface Stability, Landscape, and Molecular Orientation. *Adv. Mater. Interfaces* **2**, 1500329-1500336 (2015).

14. Han, G., Guo, Y., Duan, R., Shen, X. & Yi, Y. Importance of side-chain anchoring atoms on electron donor/fullerene interfaces for high-performance organic solar cells. *J. Mater. Chem. A* **5**, 9316-9321 (2017).

15. Yi, Y., Coropceanu, V. & Brédas, J.-L. Exciton-Dissociation and Charge-Recombination Processes in Pentacene/C-60 Solar Cells: Theoretical Insight into the Impact of Interface Geometry. *J. Am. Chem. Soc.* **131**, 15777-15783 (2009).

16. Yi, Y., Coropceanu, V. & Bredas, J.-L. A comparative theoretical study of exciton-dissociation and charge-recombination processes in oligothiophene/fullerene and oligothiophene/perylenediimide complexes for organic solar cells. *J. Mater. Chem.* **21**, 1479-1486 (2011).

17. Menke, S. M.*, et al.* Limits for Recombination in a Low Energy Loss Organic Heterojunction. *ACS Nano* **10**, 10736-10744 (2016).
